# Anchor Group Bottlebrush Polymers as Oil Additive Friction Modifiers

**DOI:** 10.1021/acsami.3c12628

**Published:** 2023-10-09

**Authors:** Andrew Kerr, Satu Häkkinen, Stephen C. L. Hall, Paul Kirkman, Paul O’Hora, Timothy Smith, Christian J. Kinane, Andrew Caruana, Sébastien Perrier

**Affiliations:** † Department of Chemistry, The University of Warwick, Coventry CV4 7AL, U.K.; ‡ Lubrizol Limited, The Knowle, Nether Lane, Hazelwood DE56 4AN, Derbyshire, U.K.; § Rutherford Appleton Laboratory, ISIS Neutron and Muon Sourcey, Didcot OX11 0QX, U.K.; ∥ Warwick Medical School, The University of Warwick, Coventry CV4 7AL, U.K.

**Keywords:** bottlebrush polymer, friction reduction, lubricant additive, RAFT polymerization, polarized neutron reflectometry

## Abstract

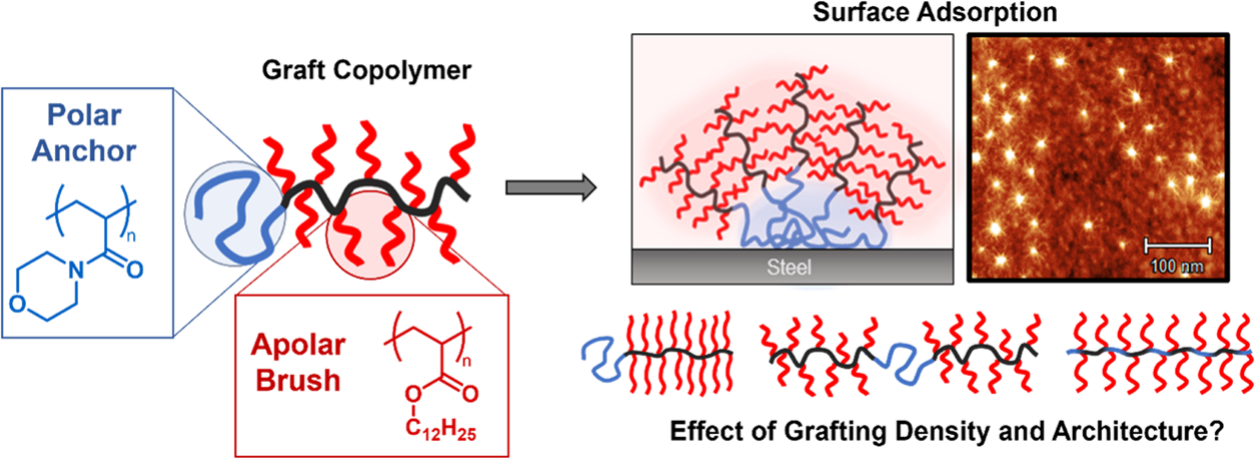

Surface-tethered polymers have been shown to be an efficient lubrication strategy for boundary and mixed lubrication by providing a solvated film between solid surfaces. We have assessed the performance of various graft copolymers as friction modifier additives in oil and revealed important structure–property relationships for this application. The polymers consisted of an oil-soluble, grafted poly(lauryl acrylate) segment and a polar, linear poly(4-acryloylmorpholine) anchor group. Reversible addition–fragmentation chain transfer polymerization was used to access various architectures with control of the grafting density and position of the anchor group. Macrotribological studies displayed promising results with ≈50% reduction in friction coefficient at low polymer treatment rates. QCM-D experiments, neutron reflectometry, small-angle neutron scattering, and atomic force microscopy were used to gather detailed information on these polymers’ surface adsorption characteristics, film structure, and solution behavior.

## Introduction

It has been estimated that one-fifth of all energy produced worldwide is used annually to overcome friction.^1^ The development and implementation of new friction-reducing technologies in road transport—such as low-viscosity and low-shear lubricants and additives—has been deemed a promising strategy to reduce energy consumption and emissions.^2^ However, thinner oils come with a trade-off, as they may be displaced from surface contacts more easily, therefore providing no lubrication or wear protection. The use of friction modifier additives is essential to meet the demand for low-viscosity lubricants and higher fuel efficiencies, making the development of new additives an important area of research.

Friction modifiers in automotive engines generally carry a polar group (such as a carboxylic acid, alcohol, amine, ester, or amide) for adsorption onto metal, and a long hydrocarbon chain to provide solubility in the base oil.^3^ Current-day friction modifiers fall under the categories of small organic molecules, such as glyceryl monooleate (GMO) and organomolybdenum compounds.^4^ These molecules bind to the surface to form a nanoscale film, which reduces friction, particularly under boundary lubrication conditions, where the distance between the surfaces is small. Alternatives to these compounds such as solid nanoparticles, polymer-coated nanoparticles, and functionalized polymers have also been explored.^5−9^ Polymer additives are an attractive subject of study in this area due to their versatile chemical and physical properties. Modern polymerization techniques have made functional polymers and their complex architectures easily accessible and attractive due to their industrial applicability.^10,11^

The lubricating effects of polymers in automotive oil formulations were noted long before they were used as friction modifiers.^12^ Polyalkyl methacrylates, commonly used as viscosity modifiers, have been found to form viscous, solvated films on metal, and to retain a fluid interface in contacts from which solvent alone would be otherwise squeezed out.^12−15^ Studies have aimed to understand the effects of polymer concentration, molecular weight, and polar groups and their placement on lubricating properties.^12,15^ Polymers in which polar groups are arranged in blocks have been found to provide better lubrication than those in which they are statistically distributed, and modeling has predicted similar effects for bottlebrush copolymers interacting with mica/silica surfaces.^16^ Block copolymers have since been the focus of many studies in both aqueous and nonpolar solvents (vide infra).

In addition to free polymer additives in the bulk phase, lubrication with surface-tethered polymers has been studied extensively.^1^ Surface modification with a polymer brush layer is known to drastically reduce friction between two sliding surfaces.^17,18^ For effective friction reduction, the selected polymer must have good solubility in the required solvent, the grafting density should be high, and the molecular weight of the grafts also impacts the performance. The dense packing of swollen polymer chains disfavors interpenetration of chains on opposing surfaces, and the entrapment of solvent molecules within the polymer layer ensures that a tribofilm is maintained even under high-pressure conditions.^19−21^ For lubrication in nonpolar environments, poly(dodecyl methacrylate) grafted onto mica has been reported to give excellent friction reduction in solvents such as hexadecane and mineral oil.^22,23^ However, covalent grafting requires prefunctionalization of a surface, whereas, in commercial applications, an oil formulation containing a friction reduction additive is more convenient and cost-effective.

Inspiration has been drawn from biological lubricants such as lubricin—a biomacromolecule found in synovial fluid, which possesses a heavily glycosylated bottlebrush-like core.^24^ Surface-tethered, densely grafted bottlebrush polymers with extended backbones and grafts have been envisioned to provide a thick, dense, and highly solvated boundary layer with limited chain interpenetration.^25^ Molecular bottlebrushes have been extensively studied for antifouling purposes and lubrication in aqueous systems,^26−28^ often to mimic the biological system of articular joints which display very low friction coefficients (μ = 0.001–0.01) over many repeat loading cycles.^29^ It has been shown that two electrostatic anchor blocks attached to a solvated bottlebrush segment provide effective surface activity; this ABA triblock bottlebrush mimicking lubricin displayed excellent lubrication performance.^30,31^ Although research in this area has mostly focused on aqueous systems, recent studies have looked at oil-soluble block copolymers with long dodecyl side groups in the oleophilic segment (akin to a bottlebrush) and a polar segment for surface adsorption.^32,33^ These additives are promising competitors for current additives in that they offer both friction and viscosity improvements from one material.^34^ ABA triblock copolymers with end groups introducing surface activity or an upper critical solution temperature have also been investigated for oil-based lubrication.^35,36^

To this end, we investigated a range of poly(lauryl acrylate) (PLA) bottlebrush architectures as friction reduction additives in oil. Reversible addition–fragmentation chain transfer (RAFT) polymerization was selected as a convenient means to synthesize complex architectures using radical polymerization. The RAFT grafting-from approach with shuttle chain transfer agent (CTA) has been demonstrated to improve control of bottlebrush synthesis, and the introduction of block copolymer segments into the backbone is facile.^37−39^ Poly(4-acryloylmorpholine) (PNAM) was selected as the polar anchor group as amide units have been shown to possess good surface activity for lubrication applications.^40^

## Results and Discussion

### Polymer Synthesis

**Figure 1:**
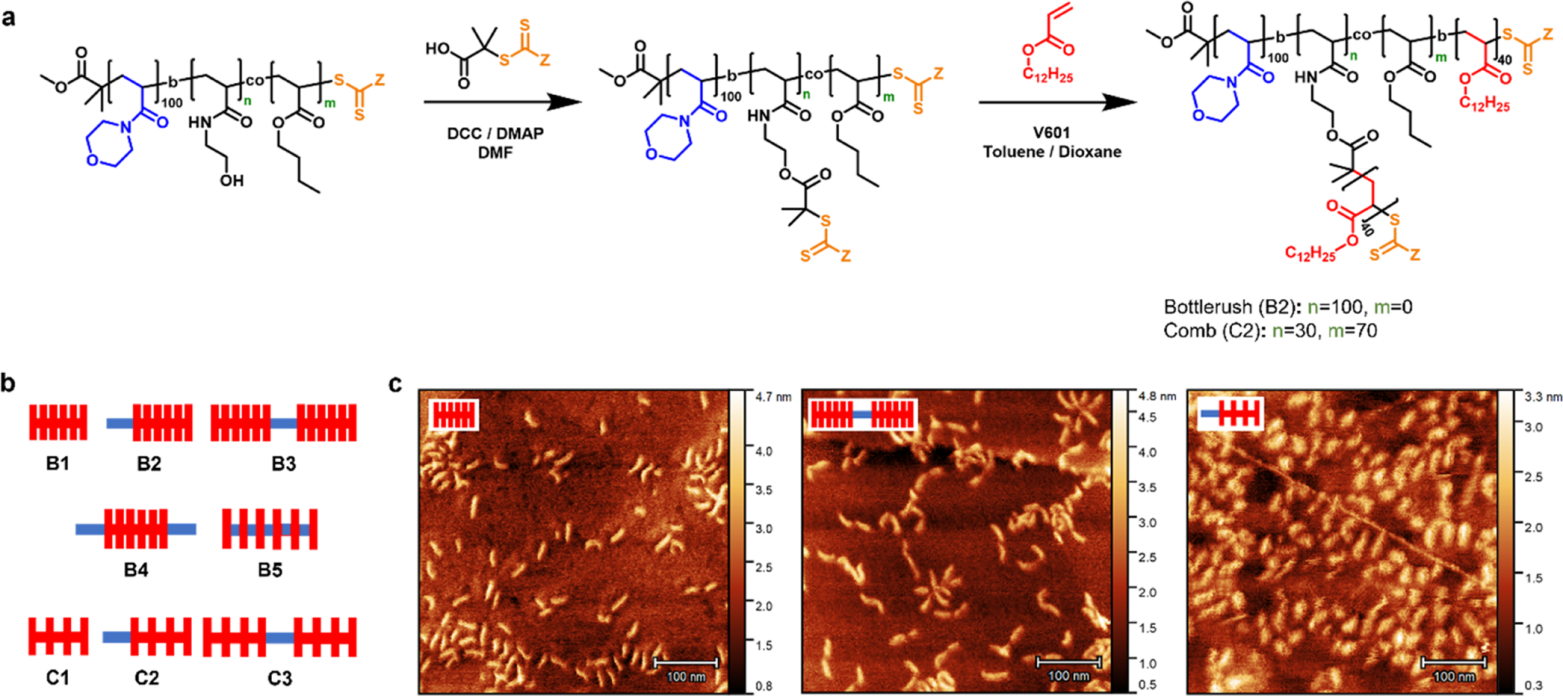
(A) Synthetic route for preparing the diblock bottlebrush (B2) and comb (C2) polymers using the RAFT grafting-from approach. (B) Schematic representation of the polymer architectures studied in this work. (C) AFM images of polymers B1 (left), B3 (middle), and C2 (right) on graphite. Scale bar = 100 nm.

The grafted architecture allows many structural parameters of a polymer to be adjusted to suit its application. These include the lengths and chemical compositions of the backbone, side chains, and anchor group; the grafting density (*n*_g_); and the positioning of grafted and linear segments. For the purposes of this study, a group of interesting architectures was selected based on literature involving noncovalently bound polymer lubricants.^30^ Previous research has identified a blocky anchor group segment to promote more effective surface adhesion than statistically distributed repeat units.^15^ Therefore, a polar linear PNAM segment was incorporated into otherwise oil-soluble grafted PLA segments (Figure 1B). High-density grafted bottlebrushes (B2–4, *n*_g_ ≈ 100%) and lower-density grafted combs (C2–3, *n*_g_ ≈ 33%) with PNAM anchors in various positions were synthesized. PLA bottlebrush and comb without an anchor (B1 and C1, respectively) and PLA bottlebrush with NAM units statistically distributed along the backbone (B5) were studied as controls. The reduced grafting density of combs was expected to increase backbone and side chain flexibility, therefore possibly influencing performance, while simplifying the synthesis as the fully grafted structures required more stringent polymerization conditions.

The graft copolymers were synthesized using the RAFT grafting-from approach (Figure 1A).^37^ The degree of polymerization (DP) of the backbones and PLA side chains was kept approximately constant (100 and 40 repeat units, respectively), and the total amount of PNAM in each structure was limited to 100 repeat units to ensure solubility in oil. This corresponded to 1.4 and 3.7 wt % polar units for the bottlebrushes and combs, respectively, in contrast with previous literature on linear polymers where approximately 10 mol % polar monomer units were found to provide effective friction reduction properties.^12^ We found such a quantity of polar NAM units to lead to insoluble materials as a result of the higher overall molecular weight.

Size exclusion chromatography (SEC) showed effective chain extension for the block copolymer backbones and relatively narrow dispersities (*Đ* ≤ 1.4) for the final products (Table 1, Figures S13–S15). Dual-angle light scattering (DALS) detectors confirmed the samples were of expected molecular weights, roughly 1 × 10^6^ g/mol for a standard brush and 4 × 10^5^ g/mol for a comb. During the graft polymerization of PLA side chains of B1–B5, free shuttle CTA was added (0.4 equiv with respect to the backbone CTAs) to improve control of the polymerization.^37^ Linear PLA chains were formed as a byproduct, corresponding to 30–42 wt % of the final material depending on the sample. While undesirable, these low-molecular-weight chains are not expected to exhibit significant surface interactions as they do not contain a polar anchor segment.

**Table 1:**
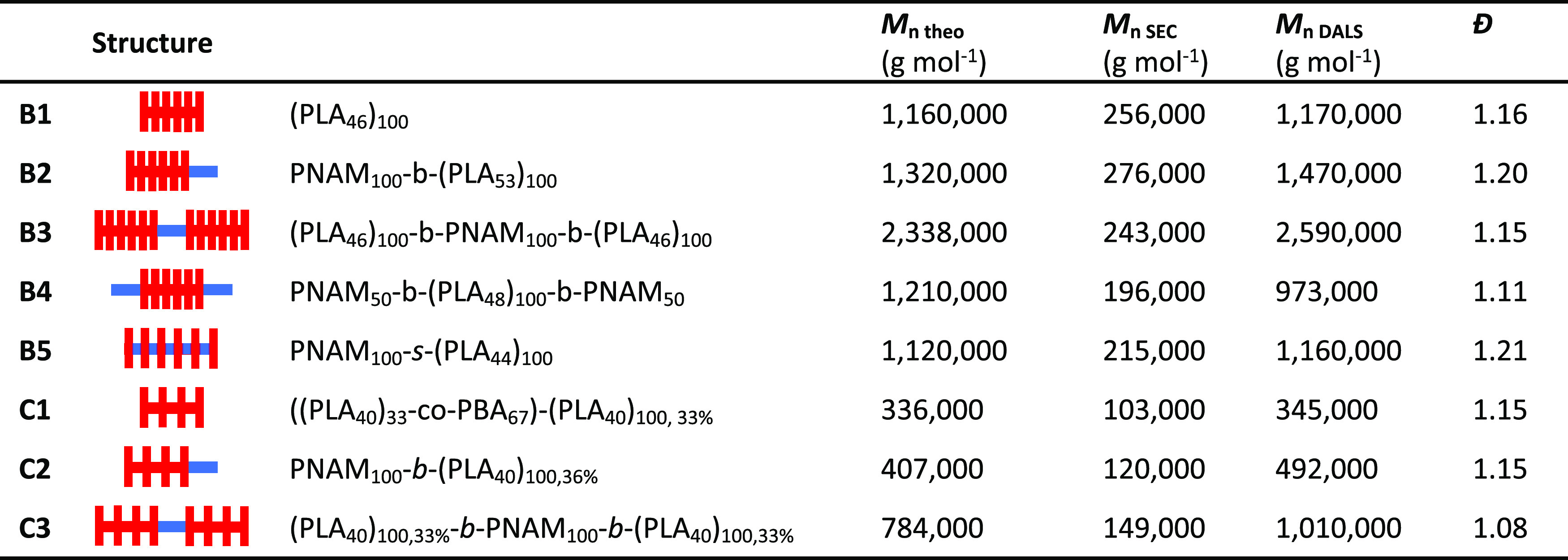
Summary of the Polymers Synthesized in This Study^a^

a SEC and DALS analyses were performed in CHCl_3_ eluent at 30 °C. *M*_n SEC_ was calculated against PMMA calibration standards and *M*_n DALS_ with the use of light scattering detectors on the SEC system.

Atomic force microscopy (AFM) imaging on graphite showed the expected cylindrical morphology for bottlebrushes B1 and B3 (Figure 1C). The images showed a contour length of approximately 25 nm (Figure S1), which was expected for a fully extended backbone with a DP of 100. For the BAB-type bottlebrush B3, the images showed a dumbbell-like structure, confirming the effective structural control achieved by the RAFT polymerization. Similar images were obtained for the combs: C1 and C2 showed an average contour length of 18 and 23 nm, respectively, which seemed reasonable for more flexible backbones due to the reduced grafting density.

### Surface Adsorption Characteristics

The lubricant performance of these polymers was initially hypothesized to primarily depend on their surface adsorption characteristics, which in turn was anticipated to be dependent on the position of the PNAM segment. To assess the role of its incorporation and placement, surface adsorption of the polymers and the rigidity of the resulting films were studied using a quartz crystal microbalance with dissipation (QCM-D; see the Supporting information for experimental details).

**Figure 2:**
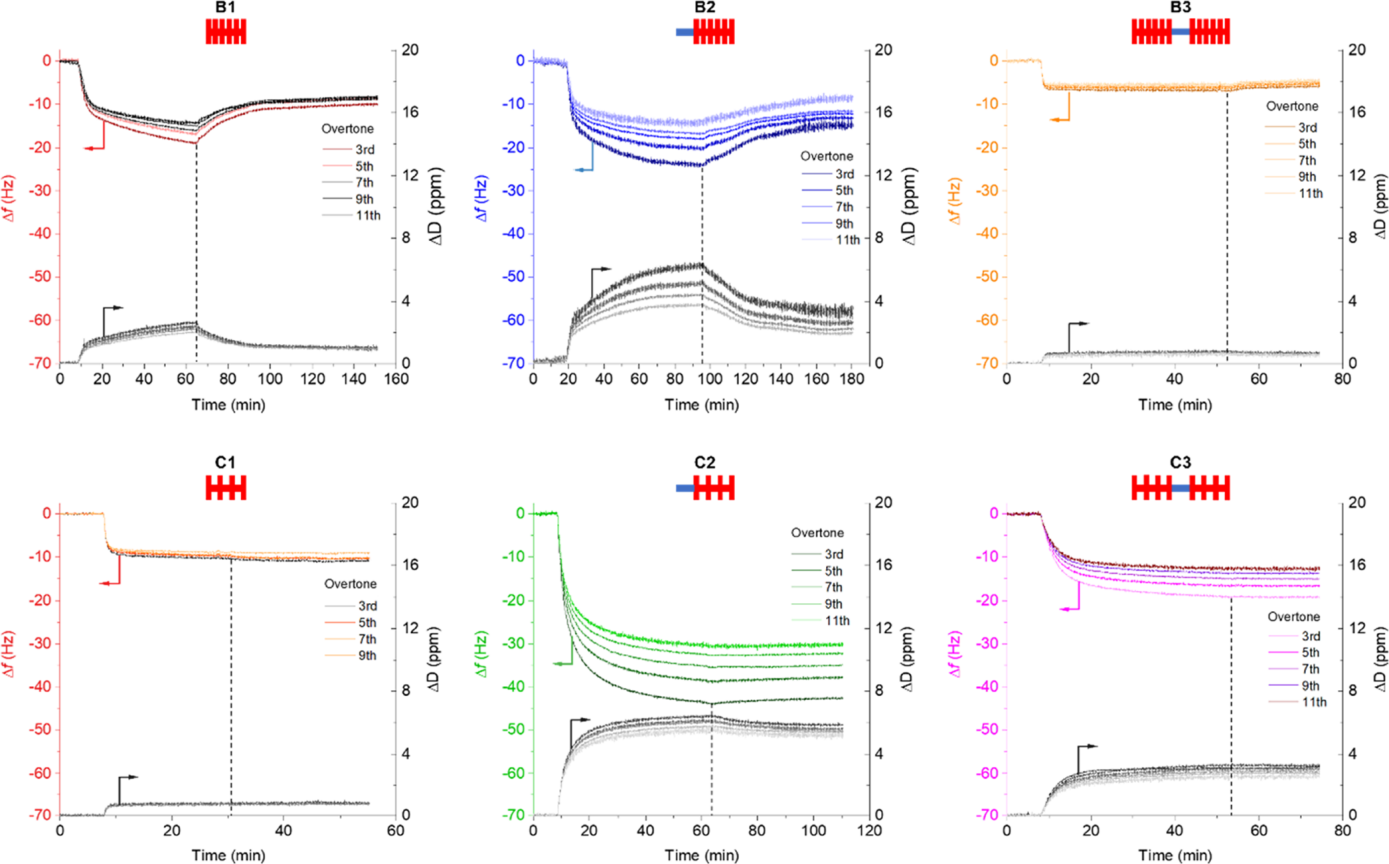
QCM-D data were collected for the adsorption of the brush and comb copolymers from *n*-dodecane onto steel. The starting point of rinse with pure solvent is indicated by a dashed line.

Solutions of 0.01–0.1 wt % polymer in *n*-dodecane were passed over a stainless steel-coated chip at a 50 μL/min flow rate at 40 °C. The data showed a sharp decrease in the QCM sensor resonance frequency for each polymer after injection, arising from mass deposition onto the steel surface (Figure 2). After a plateau was reached, the system was rinsed with pure *n*-dodecane to remove any loosely adsorbed polymer. The rinse was found to have very little effect on the resonance frequency of the combs, suggesting that all of the adsorbed polymer was tethered to the surface tightly enough to not desorb at the selected flow rate. However, a significant amount of bottlebrushes B1–2 were removed in this step, possibly due to washing of the low molecular weight linear polymer side product. Each polymer showed distinct adsorption behavior in both the magnitude of frequency change and overtone splitting, indicating differences in surface activity and the viscoelasticity of the resulting films.

Unfunctionalized samples B1 and C1 displayed some surface adsorption which likely arises from interactions of polar ester/amide units with the surface, as has previously been described for poly(alkyl methacrylate) derivatives.^33^ A comparison of Δ*f* (frequency change) between bottlebrushes B1 and B2 and combs C1 and C2 confirmed the incorporation of a PNAM segment into the polymer structure to result in increased surface adsorption. Polymers in which the PNAM segment was flanked by two grafted segments (B3 and C3) exhibited a smaller Δ*f* than their diblock counterparts (B2 and C2), suggesting that increased steric shielding of the central anchor block may inhibit effective surface interaction. It is worth noting that the molar mass of B3 is roughly double that of B2, meaning that the difference in the number of adsorbed molecules is even greater than the absolute difference in the Δ*f* vs *t* plot.

Reduced grafting density of C2 and C3 resulted in a significant increase in adsorption when compared to that of their densely grafted counterparts B2 and B3. Interestingly, C3 exhibited adsorption greater than that of bottlebrush B3. This could be attributed to the increased flexibility of the grafted segment and reduced steric hindrance around the PNAM anchor, allowing the latter to interact better with the surface. Polymers C2 and C3 showed significant overtone splitting, indicative of a viscoelastic film.

### Lubricant Performance

The friction reduction performance of the synthesized polymers was assessed with a mini traction machine (MTM) across a 40–140 °C temperature range in 20 °C increments at 1 wt % treatment rate. At this relatively low concentration of additive, polymers showed a negligible increase in the base oil viscosity (Table S2). Due to the presence of a linear byproduct in these samples, the effective treatment of graft copolymer actives ranged from 0.6 to 0.9 wt %. The MTM measures the traction between two lubricated surfaces in relative motion by rotating a steel ball loaded against a rotating steel disk. The speeds of the ball and disk are controlled independently which enables a continuum from pure rolling (0% slide-to-roll ratio/SRR) to pure sliding (200% SRR). The Stribeck curves were produced by measuring friction as a function of speed under constant load and temperature. The speed range that the data points were taken over was 3000–10 mm/s, representing the transition between EHD and boundary lubrication regimes. Viscosity has an impact on traction measurements in the EHD regime; hence, the fluid viscosities must be closely aligned for comparison in this region. In the boundary regime, the fluid viscosity plays little part in dictating the traction which is dominated by surface effects.

**Figure 3:**
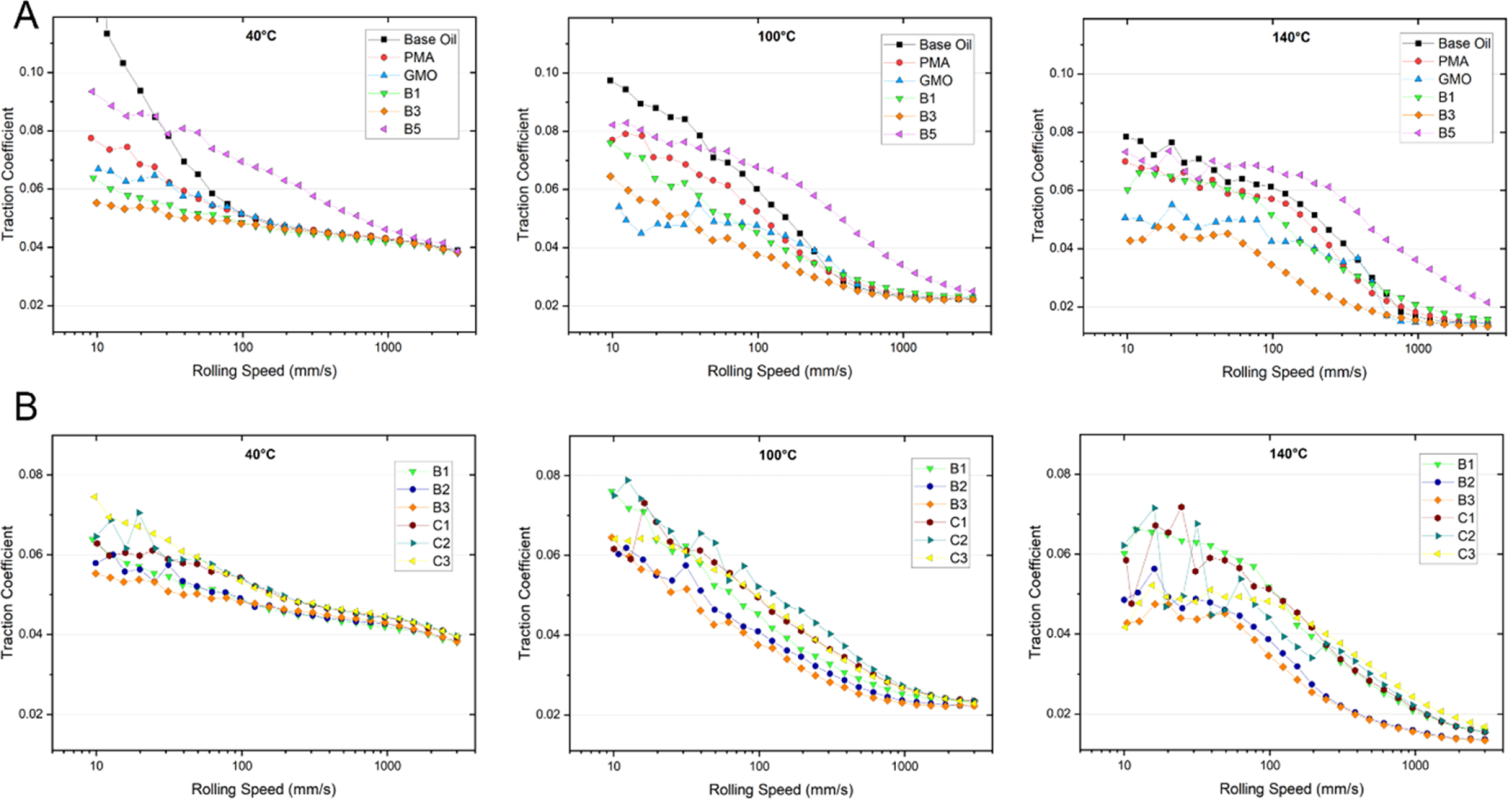
MTM testing of Yubase 4 mineral oil treated with ≤1 wt % actives. (A) Comparison between plain base oil and treated oils containing synthesized bottlebrush polymer, unfunctionalized linear polymethacrylate (PMA), or glyceryl monooleate (GMO). (B) Comparison between the bottlebrush and comb samples. The frictional force was described by Stribeck curves in which the traction coefficient is plotted against the so-called Hersey number (*x*), which is given by *x* = *ηv/N*, where η is the viscosity, *v* is the entrainment speed, and *N* is the normal load. Under a constant load and viscosity, the traction coefficient may be plotted as a function of the entrainment speed.^42^

A comparison was first made between plain base oil and treated oil containing a bottlebrush polymer additive synthesized herein, a commercially used organic friction modifier glyceryl monooleate, or a commercial unfunctionalized viscosity modifier polyalkylmethyl acrylate (PMA) (Figure 3A). In all cases, the presence of a polymer additive leads to a substantial decrease in friction coefficient at slow rolling speeds and elevated temperatures. The poor performance for the statistical brush was in line with expectations from previous literature, as statistically distributed polar units may more readily detach from the surface, disfavoring film formation.^15^ However, the observed increase in friction compared with base oil was unexpected. This can be explained by adhesive bridging effects arising from intermolecular interactions of the randomly distributed PNAM units between brush molecules on opposing surfaces.^41^

Bottlebrush B1 showed slightly improved performance compared to that of the linear PMA, although both are quite poor friction modifiers. Weakly interacting bottlebrushes (i.e., those without an anchor group) may be more effective than linear polymers as a result of the increased molecular size. Good performance was found for the triblock brush B3, reducing friction by ≈50% at 120 °C and 10 mm/s rolling speed with respect to the base oil, and outperforming GMO. The introduction of the anchor group evidently is essential for good friction reduction performance as demonstrated by the comparison of B2 and B3 vs the unfunctionalized B1 (Figure 3B). The performance of the anchored polymers was strongly temperature-dependent: an increasingly large improvement was seen with respect to hydrophobic polymers (PMA, PLA brush) with an increasing temperature (Figure S16). The reduction in oil viscosity at elevated temperatures can shift the boundary lubrication regime toward higher rolling speeds which explains the increased effect of the lubricants.^43^

A comparison was then made between densely grafted brushes and lower-density combs (Figure 3B). All combs provided a 20–30% reduction in the friction coefficient in the boundary regime at 40 °C and a larger reduction of 30–50% at 140 °C with respect to the base oil. The presence of a PNAM segment was not found to have as large of an effect on the performance of the combs.

It is worth noting that the ratio of molecular weights of the polar anchor group and the solvophilic graft segments is quite different between the bottlebrush and comb samples, which could have a strong effect on the solubility and aggregation behavior in oil. The increased number of PLA units in the triblock comb and brush samples could improve solubility sufficiently such that they are more able to adsorb to the surface, improving performance.

A noticeable feature in the data was the ability of anchor group brushes to retain lower friction coefficients than all of the other compounds—including GMO—at intermediate rolling speeds (100–500 mm/s) and high temperatures. Organic friction modifiers such as GMO are generally believed to form monolayers on the steel surface (typically < 2 nm),^44^ whereas bottlebrush polymers could form much thicker films of ∼20 nm that may better support the load and prevent direct surface contact. These well-solvated bottlebrush films can retain oil among the PLA side chains, resulting in thicker tribofilms and preventing entry into the mixed and boundary regimes until slower rolling speeds. This suggests that the bottlebrushes form thicker films than combs under the MTM conditions, although this is in contrast with the QCM-D data, which showed a larger mass deposition for the combs. It is worth noting the differences in temperature and shear conditions between the two techniques which could explain such discrepancies, the harsher conditions under MTM testing may more readily desorb nonfunctionalized and weakly interacting species. Second, the difference in grafting density of the combs and brushes could explain the differing lubrication performance. For covalently surface-grafted polymers, higher density of chain packing is known to reduce friction as a result of excluded volume effects reducing entanglements between opposing polymer-covered layers.^45^ It has been predicted that branched dendronized surfaces will show lower interpenetration distances under compression than standard linear surface-grafted systems.^46^ The high-density bottlebrushes studied here have a more branched topology than the combs, which accordingly should also lead to a shorter interpenetration distance. Therefore, a film derived from rigid bottlebrushes may be expected to lubricate more effectively than a film consisting of its less rigid comb equivalent even if lower quantities are adsorbed on the surface. This is consistent with the generally superior performance of the brush materials in MTM, although the good friction reduction of the triblock comb suggests this may not be so significant.

### Film Structure and Solution Behavior

QCM-D data showed some of the tested polymers readily adsorb onto steel, forming a viscoelastic film. However, surface coverage, film thickness, and polymer orientation on the surface remained unknown and have been identified as key parameters for high performance in some boundary lubricants.^47^ Rigid bottlebrush polymers could be envisioned to adopt a flat, perpendicular, or tilted orientation on the surface, all of which would result in different layer thicknesses. The layer structure could be further complicated by micelle formation in the bulk phase. Small-angle neutron scattering (SANS) of polymer solutions (6 mg/mL) prepared in *n*-dodecane-*d*_26_ suggested combs C2–3 to form micelles, whereas brushes B1–5 and unfunctionalized comb C1 appeared to exist as unimolecular species in the solution (Figure S2).

QCM-D data showed that the architecture that appeared to give the highest degree of adsorption was the diblock comb (C2). We therefore chose to focus on this compound as a model system to investigate the structure of these polymer films using polarized neutron reflectometry (PNR). The sample of interest consisted of a solvated layer of polymer adsorbed onto a solid steel substrate, immersed in *n*-dodecane. The substrate comprised a silicon block polished to <5 Å roughness and sputter-coated first with permalloy (4:1 Ni/Fe) and then grade 316 stainless steel. The magnetic layers of the substrate provided an additional spin contrast to complement the isotopic solvent contrast when measured with spin-polarized neutrons. The measurement setup consisted of a polarized neutron beam directed at the sample within a laminar flow cell maintained at a 45 °C temperature, to which pure solvent or polymer solution could be injected using a syringe pump. Measurements were carried out at 0.5, 1.5, and 2.5° incident angles to cover an effective *Q*_z_ range of 0.01–0.03 Å^–1^ using spin-up (↑) and spin-down (↓) polarized neutrons and two isotopic solvent contrasts, hydrogenated and deuterated *n*-dodecane.

The clean substrate was first measured to give four reflectivity profiles describing the solid–solid interfaces within the substrate and the solid–liquid interface between the substrate and the solvent (Figure S3). Four layers were required to fit these data, corresponding to SiO_2_, permalloy, steel, and a thin oxide layer (Table 2), the parameters of which were later fixed for the analysis of the polymer film. The fits were in good agreement with the expected 150 and 250 Å thicknesses of permalloy and steel, respectively. Due to the poor contrast of the oxide layer against the hydrogenated solvent, a high level of uncertainty remained in its fitted parameters, most notably with the scattering length density (SLD) and solvation, which are intrinsically correlated.

**Table 2:** Structural Parameters Obtained from Fitting PNR Data for a Silicon–Permalloy–Steel Substrate before and after Incubation with PNAM_100_-*b*-(PLA_40_)_100,36%_ (C2)^a^

layer	SLD (×10^–6^ Å^–2^)	thickness (Å)	solvation (%)	roughness (Å)
Si	2.07*		0*	4_–1_^+2^
SiO_2_	3.47*	30_–2_^+2^	0*	11_–1_^+1^
permalloy ↑	10.19_–0.03_^+0.03^	137_–1_^+1^	0*	10_–1_^+1^
permalloy ↓	6.73_–0.03_^+0.03^			
steel	6.27_–0.02_^+0.03^	260_–2_^+1^	0*	11_–1_^+1^
oxide	0.36_–0.35_^+0.65^	15_–2_^+2^	7.0_–7_^+9^	9_–1_^+1^
PNAM	1.26*	47_–7_^+7^	67_–4_^+4^	19_–2_^+1^
PLA	0.14*	156_–22_^+21^	95_–1_^+1^	16_–10_^+4^
*n*-dodecane	–0.53_–0.03_^+0.07^			
*n*-dodecane-*d*_26_	5.94_–0.01_^+0.01^			

a The substrate was first characterized with both solvent contrasts using spin-up (↑) and spin-down (↓) neutrons. Fitted parameters marked with asterisks were then fixed for analysis after incubation with polymer. Error values were calculated from the 95% confidence intervals estimated from the Markov chain Monte Carlo analysis.

**Figure 4:**
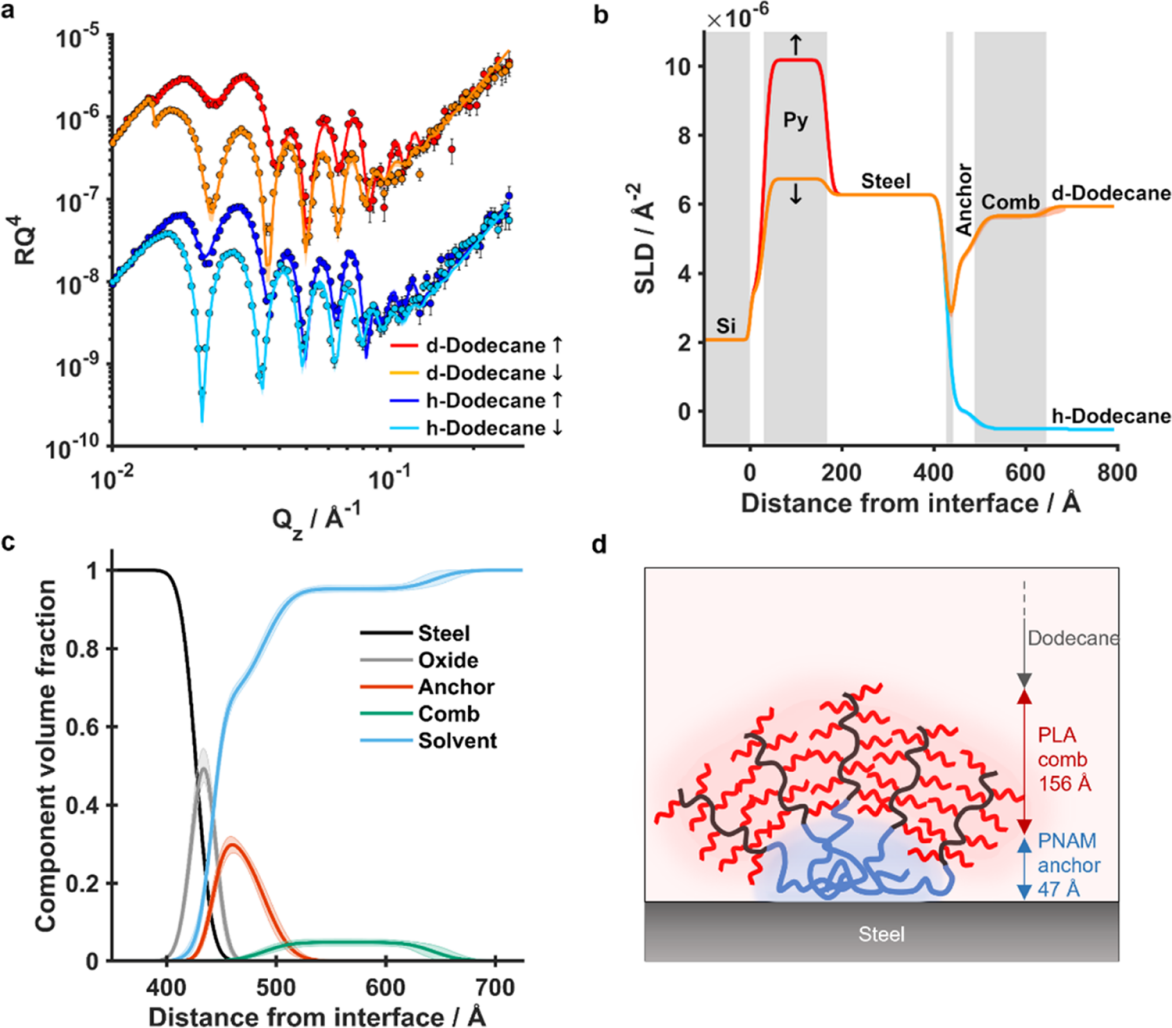
Polarized neutron reflectometry of comb polymer (C2) film on steel. (A) Data (points) and fits (lines) plotted as RQ^4^ for Si–Py–Steel substrates with adsorbed PNAM_100_-*b*-(PLA_40_)_100,36%_, characterized in h- and d-dodecane isotopic contrasts with spin-up (↑) and spin-down (↓) magnetic contrasts. Data and fits corresponding to d-dodecane isotopic contrasts have been vertically offset for clarity. (B) SLD profiles corresponding to the fits shown in (A). Gray-shaded regions indicate discrete layers included in the model. Colored, shaded regions indicate the 95% confidence interval associated with the fit as determined by MCMC. (C) Component volume fraction profile. (D) Schematic illustration of data interpretation.

To measure the polymer layer, 0.1% w/v polymer solution in *n*-dodecane was injected into the flow cell at a 0.5 mL/min flow rate and incubated for 2 h, after which pure *n*-dodecane was passed through to remove any weakly bound polymer and that remaining in the bulk phase. For these data, an additional two layers were required to fit the data, corresponding to the linear PNAM and grafted PLA segments (Figure 4). The fits were in good agreement with the experimental data, showing a total film thickness of roughly 20 nm, consisting of a 5 nm PNAM layer and a 16 nm PLA layer. The thickness and roughness of the former were much higher than would be expected of a linear polymer lying flat on a surface, suggesting the PNAM segments adopt a different orientation. Considering that SANS suggested micellization of this polymer, it seems likely that the layer consists of aggregated PNAM chains, covering roughly 30% of the surface as suggested by the degree of solvation. The surface coverage may be limited by the steric constraints of the grafted PLA segment. The thickness of the PLA layer corresponds to roughly 60% of the length of a fully extended backbone, a reasonable value for the sparsely grafted chain. The 10% roughness of this layer suggests different chain orientations for the grafted segment. No off-specular intensity was observed in the data, suggesting that if clustering was occurring, it was showing no long-range order or regularity. It can be hypothesized that most combs are clustered together as diffuse micelles on the substrate, while some may exist as individually adsorbed molecules.

**Figure 5:**
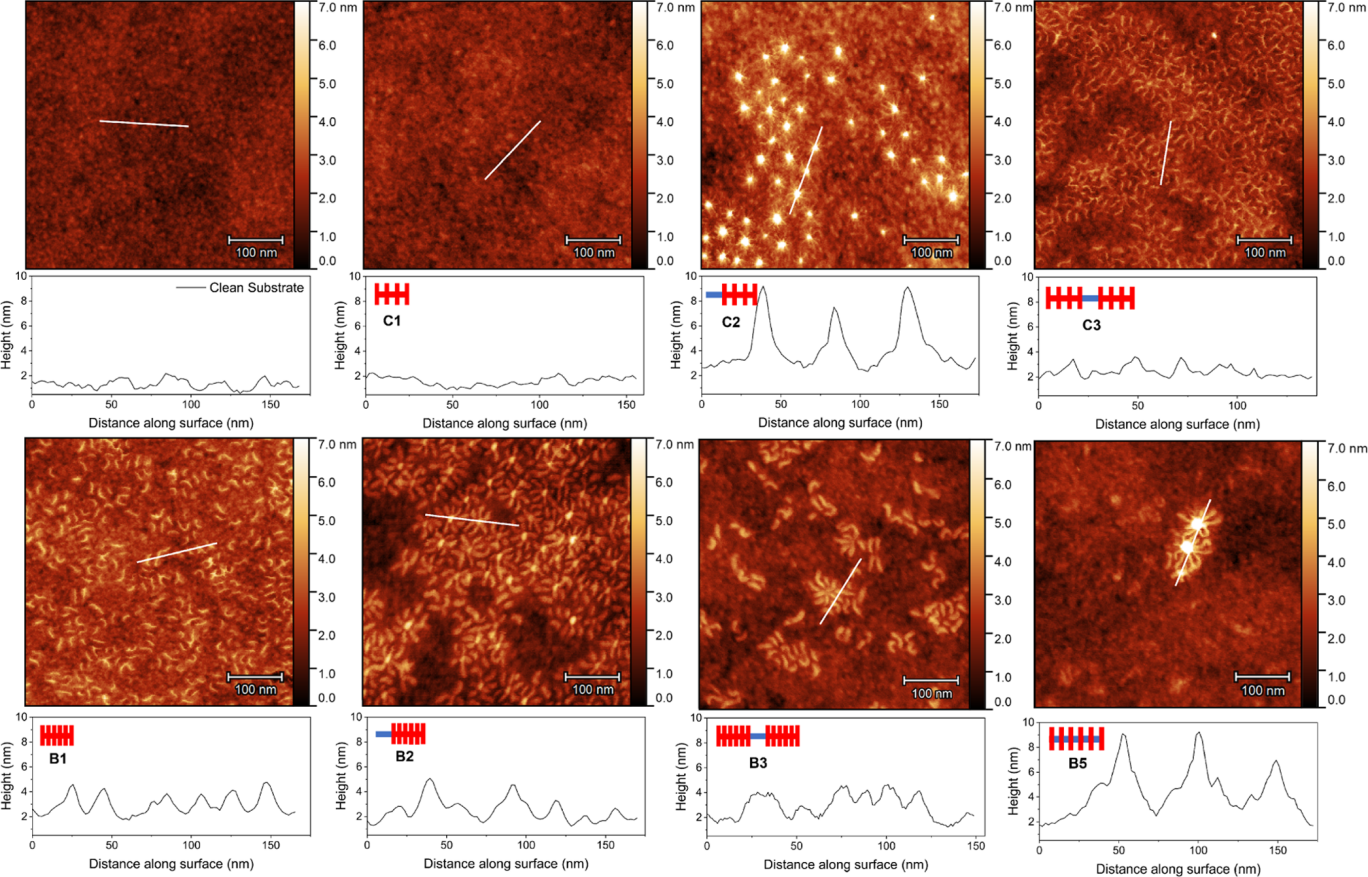
AFM images of PNR steel substrates after incubation in a polymer solution prepared in *n*-dodecane. The samples were rinsed with pure solvent after incubation to remove loose polymer, dried, and measured under ambient atmosphere.

AFM images of polymers on the PNR substrates were collected in the dry state after mimicking the adsorption conditions of the PNR experiment including polymer incubation and rinsing with solvent. The images revealed significant differences across the studied architectures (Figure 5). Anchored combs C2 and C3 were found on the surface in abundance; however, no polymer was found on the substrate for unfunctionalized comb C1. This was in agreement with QCM-D data, in which low mass deposition was measured after rinse for the latter, while C2 and C3 remained in larger quantities. Furthermore, images of C2 revealed the presence of star-like micelles with a dense PNAM core surrounded by grafted PLA segments extending radially outward, corroborating the PNR and SANS results. These assemblies of C2 were large in height profile (∼7 nm) compared to the other samples, although they had a lower total surface coverage area compared to B2 and C3. These micellar assemblies may explain the high mass deposition measured by QCM-D for C2, but the inconsistent surface coverage leads to a lackluster friction reduction. The PNAM segments of C3 also formed intermolecular aggregates; however, the apparent aggregation number (*n*_agg_ ≈ 3–4) was much lower than for C2, likely due to increased steric hindrance.

Bottlebrushes B1–B3 were readily adsorbed onto the substrate with coverage densities roughly correlating to the thicknesses observed in QCM-D. Unlike comb C1, the unmodified brush B1 retained a surface layer, possibly due to the increased molar mass and differences in the backbone chemistry. Compared with C1, B1 contains a larger number of backbone amide units derived from the HEAm monomer, which could promote stronger surface adsorption.

The PNAM anchor group also induced the formation of star-like micellar species for B2–3, although the aggregation number is much lower than for the combs and a number of individually absorbed brush molecules are still apparent. This may be expected from the increased steric bulk of the more rigid brush segment, reducing the freedom of the PNAM anchor. In addition, B5 revealed very low surface coverage with larger, poorly defined flower-like micellar structures, which could explain its poor lubrication performance.

Overall, anchor group bottlebrushes and combs seemed superior in forming uniform films, showed the most consistent data in friction tests, and could be considered the most promising candidates for further testing.

## Conclusions

A variety of complex graft copolymer architectures were prepared and assessed as boundary-film-forming lubricants in oil to guide the design of next-generation friction modifier additives. RAFT polymerization enabled the synthesis of highly defined PLA graft copolymers with polar PNAM anchor groups selectively incorporated into different positions of the polymer architecture.

QCM-D experiments showed that the incorporation of a PNAM anchor segment significantly increased mass deposition onto a steel surface, while the overall architecture also had a large influence. Central PNAM anchor blocks reduced surface absorption with respect to end-grafted anchors, while loosely grafted combs produced more viscoelastic films and absorbed more abundantly than densely grafted bottlebrushes. Translation of these surface properties to friction performance was not clear, however, with bottlebrushes displaying slightly improved lubrication over the combs in MTM testing, but otherwise generally similar performance was observed across the different architectures. AFM studies indicated substantial differences in adsorbed polymer morphology, whereby the addition of the polar anchor group induced assembly into star-like micelles and absorption of said aggregates onto the surface. The diblock comb C2 formed assemblies with a high aggregation number, leading to thick (20 nm) films that could be successfully analyzed by PNR to elucidate the micellar composition, revealing the interaction of the PNAM block directly with the surface.

The structure–property relationships in nonpolar systems studied here may be used to guide the future design of polymers with improved performance. Aside from the polymer architecture, optimization could involve changes in the side chain length, backbone length, grafting density, or selection of the polar anchor group. Finally, the correlation of additive performance to film structure could be made more reliable by employing instruments that may be used to monitor film formation in situ under shear.

## Supporting Information Available

Additional experimental details, materials, and methods (PDF)
