# Supplementary material for: Anchor Group Bottlebrush Polymers as Oil Additive Friction Modifiers
Source: ACS Appl Mater Interfaces. 2023 Oct 9;15(41):48574–83. doi: 10.1021/acsami.3c12628 (PMC10591277; doi:10.1021/acsami.3c12628)
Supplement: Supplementary file 1 — am3c12628_si_001.pdf [file am3c12628_si_001.pdf]

Supporting Information

Anchor Group Bottlebrush Polymers as Oil Additive Friction Modifiers

Andrew Kerr[1], Satu Häkkinen[1], Stephen C. L. Hall[1], Paul Kirkman[2], Paul O'Hora[2], Timothy Smith[2] Christian J. Kinane[3], Andrew Caruana[3], Sébastien Perrier[1,4]*

[1] Department of Chemistry, The University of Warwick, Coventry CV4 7AL, U.K

[2] Lubrizol Limited. The Knowle, Nether Lane, Hazelwood, Derbyshire DE56 4AN. UK

[3] ISIS Neutron and Muon Source, Rutherford Appleton Laboratory, Didcot, OX11 0QX, UK

[4] Warwick Medical School, The University of Warwick, Coventry CV4 7AL, U.K.

* Correspondence to s.perrier@warwick.ac.uk

Table of Contents

1 Materials 3
2 Instrumental methods 4
2.1 Nuclear Magnetic Resonance (NMR) Spectroscopy 4
2.2 Mini Traction Machine (MTM) Testing 4
2.3 Quartz Crystal Microbalance with Dissipation (QCM-D) 4
2.4 Atomic Force Microscopy (AFM) 5
2.5 Size Exclusion Chromatography (SEC) 5
2.5.1 DMF SEC 5
2.5.2 CHCl3 SEC 6
2.5.3 THF SEC 6
2.6 Small-Angle Neutron Scattering (SANS) 6
2.7 Polarised Neutron Reflectometry (PNR) 9
2.8 Viscosity Measurements 14
3 Synthetic protocols 15
3.1 Synthesis of di-BIBDTC 15
3.2 Polymerisation of backbones for the mono- and diblock structures 17
3.2.1 Backbone synthesis for densely grafted brushes B1, B2 and B5 17
3.2.2 Backbone synthesis for loosely grafted combs C1 and C2 18
3.3 Polymerisation of backbones for the triblock structures 19
3.3.1 Backbone synthesis for densely grafted brush B4 19
3.3.2 Backbone synthesis for densely grafted brush B3 20
3.3.3 Backbone synthesis for loosely grafted comb C3 20
3.4 Functionalisation of backbones 21
3.4.1 Densely grafted brushes 21
3.4.2 Loosely grafted combs 22
3.5 Polymerisation of PLA side chains 24
3.5.1 Densely grafted brushes 24
3.5.2 Loosely grafted combs 28
4 References 32

1 Materials

4-Acryloylmorpholine (NAM, 97%), lauryl acrylate (LA, 90%), butyl acrylate (BuA, ≥99%) and *N*-hydroxyethyl acrylamide (HEAm, 97%) were obtained from Sigma-Aldrich and passed through a basic alumina column before use. Dimethyl sulfoxide-d_6 (DMSO-d_6, 99.9% D atom), chloroform-*d* ($CDCl_3$, 99.8% D atom), deuterium oxide (D_2O, 99.9% D atom), methanol, dichloromethane (DCM, anhydrous) and oxalyl chloride (2.0 M in methylene chloride) were obtained from Sigma-Aldrich and used as received. 4-(Dimethylamino)pyridine (DMAP, ≥99.0%) and *n*-dodecane (99+%) were obtained from Alfa Aesar and used as received. 1,4-Dioxane, toluene and *N*,*N*-dimethylformamide (anhydrous) were obtained from Fisher Scientific and used as received. *N*-(3-Dimethylaminopropyl)-*N*′-ethylcarbodiimide hydrochloride (EDC) was obtained from Carbosynth and used as received. RAFT agents 2-(((butylthio)carbonothioyl)thio) propanoic acid (PABTC), methyl 2-(((butylthio)carbonothioyl)thio)propanoate (PMBTC) and ethane-1,2-diyl bis(2-(((butylthio)carbonothioyl)thio)propanoate) (DiPABTC) were synthesised according to literature procedures.[1, 2] Initiators lauroyl peroxide (LPO, 97%, Aldrich), 2,2′-azobis[2-(2-imidazolin-2-yl)propane]dihydrochloride (VA-044, >98%, Wako) and dimethyl 2,2’-azobis(2-methylpropionate) (V-601, >98%, Wako) were used as received. *n*-Dodecane-d_{26} (d-dodecane, 98%) was purchased from Cambridge Isotope Laboratories (UK).

Single crystal silicon substrates used in PNR experiments were purchased from Crystran Ltd (UK). The substrates were 80 × 50 × 15 mm in size with a single 80 × 50 Si(111) face polished to < 5 Å root-mean-square roughness. The surface was sputter-coated with permalloy (4:1 Ni:Fe) and 316-type steel films of 150 and 250 Å thickness, respectively, by the NanoFab group at the Center for Nanoscale Science and Technology, NIST (USA).

2 Instrumental methods

2.1 Nuclear Magnetic Resonance (NMR) Spectroscopy

^{1}H and ^{13}C NMR data were collected on Bruker Avance 300 MHz, Bruker Avance III HD 300 MHz and Bruker Avance III 400 MHz spectrometers at 300 K in deuterated solvents (DMSO-d_6, $CDCl_3$ or D_2O). Chemical shifts (δ) are reported in parts per million (ppm). Residual solvent signals were used for calibration.

2.2 Mini Traction Machine (MTM) Testing

Stribeck curves were measured using a PCS Instruments mini traction machine (MTM2) fitted with a ball and disc pair (PCS Instruments Standard Specimen Pack) made from AISI 52100 alloy steel with surface finish <0.02 µm Ra. A new ball (19 mm) and disc (Ø=46 mm) were fitted for each sample. Performance tests were conducted with 1 wt.% additive in Yubase 4 mineral oil. The slide-to-roll ratio was fixed at 50%, the load at 37 N, and the contact pressure at 1.0 GPa. Data was collected at 40-140 °C sequentially using the same test components over a speed range of 10-3000 mm/s. Each sample was tested only once due to limited sample size.

2.3 Quartz Crystal Microbalance with Dissipation (QCM-D)

QCM-D adsorption experiments were carried out at Lubrizol using a QSense E4 system equipped with a peristaltic pump (IPC-C, Ismatec), solvent resistant tubing, and flow cells with solvent resistant gaskets and O-rings. QSense stainless steel-coated (grade SS2343) QCM chips were used for the analysis. Solvent and solutions were filtered using Acrodisc Supor PES syringe syringe filters with 0.2 µm pore size and 32 mm diameter. Prior to the experiment, the chip was cleaned by sonicating in toluene for 10 min, drying under N_2, soaked in Hellmanex solution (1 wt% in deionised water) for 30 min, rinsing with deionised water, dried under N_2, sonicating in ethanol for 10 min and drying under N_2. Finally, the chips were placed in an ozone cleaner for 10 min. The chips were loaded into cells set to 40 °C and pure dodecane was passed through the sample cell at a flow rate of 50 µl/min until a stable frequency reading was observed. The sample (0.1 or 0.01 wt% in *n*-dodecane) was then passed over the chip using the same flow rate until the frequency reading reached a plateau. Pure dodecane was then injected to rinse the system. Frequency and dissipation changes were monitored for all tuned overtones with data collection controlled by QSoft software. Data analysis was performed with QTools software.

2.4 Atomic Force Microscopy (AFM)

For imaging on highly oriented pyrolytic graphite, samples were prepared by drop casting a 0.05 mg/ml polymer solution in *n*-hexane (brushes) or chloroform (combs) onto a freshly cleaved substrate and dried under a gentle N_2 flow. For imaging on steel, the substrates were cleaned prior to use in the same manner as described for the neutron reflectometry. Samples were prepared by submerging the steel substrate in a polymer solution prepared in *n*-dodecane (0.1 wt%) for 30 min. The polymer solution was displaced with pure *n*-dodecane to remove excess sample while keeping the substrate submerged. The substrate was then placed in a beaker of pure *n*-hexane (x2) and left to dry for ≥ 10 min before analysis

Images were collected using a Bruker Dimension Icon instrument with ScanAsyst in Air and PeakForce tapping. Images were processed with Gwyddion software.

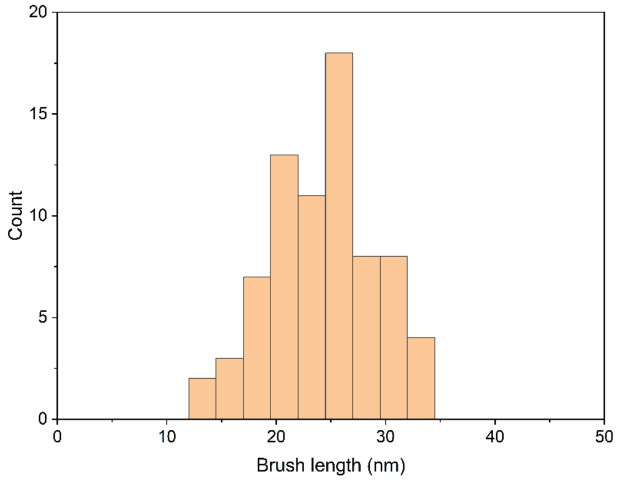

Figure S1. Histogram of B1 brush molecule backbone lengths measured by analysis of AFM images using Gwyddion software. Mean length = 24.2 nm, STD = 4.7 nm.

2.5 Size Exclusion Chromatography (SEC)

Experimental molar mass ($M_{\text{n SEC}}$) and dispersity ($Đ$) values of synthesised polymers were determined by employing conventional calibration with poly(methyl methacrylate) standards (Agilent EasyVials) or DALS using Agilent GPC/SEC software.

2.5.1 DMF SEC

Analysis was carried out on an Agilent Infinity II MDS instrument equipped with differential refractive index (DRI), viscometry (VS), dual angle light scatter (DALS), and single-wavelength UV detectors, 2 x PLgel Mixed D columns (300 x 7.5 mm; 200 to 400,000 g/mol linear operating range for PS equivalent) and a PLgel 5 µm guard column. The eluent

was DMF with 5 mmol NH_4BF_4 additive. Analyte samples were filtered through a nylon membrane with 0.22 μm pore size before injection. Samples were run at 1 ml/min at 50 °C.

2.5.2 CHCl3 SEC

Analysis was carried out on an Agilent Infinity II MDS instrument equipped with differential refractive index (DRI), viscometry (VS), dual angle light scatter (DALS), and multi-wavelength UV detectors, 2 x PLgel Mixed C columns (300 x 7.5 mm; 200 to 2,000,000 g/mol operating range for PS equivalent) and a PLgel 5 µm guard column. The eluent was $CHCl_3$ with no additives. Analyte samples were filtered through a PTFE membrane with 0.22 μm pore size before injection. Samples were run at 1 ml/min at 30 °C.

2.5.3 THF SEC

Analysis was carried out on an Agilent Infinity II MDS instrument equipped with differential refractive index (DRI), viscometry (VS), dual angle light scatter (DALS), and multiple-wavelength UV detectors, 2 x PLgel Mixed C columns (300 x 7.5 mm; 200 to 2,000,000 g/mol operating range) and a PLgel 5 µm guard column. The eluent was THF with 0.01 % butylated hydroxytoluene additive. Analyte samples were filtered through a PTFE membrane with 0.22 μm pore size before injection. Samples were run at 1 ml/min at 30 °C.

2.6 Small-Angle Neutron Scattering (SANS)

Polymer solution (6 mg/ml in *n*-dodecane-$^2H_{26}$) was loaded into rectangular quartz cuvettes with a 1 mm path length. Data were collected after sample equilibration at 40 °C using the LARMOR instrument at the ISIS Neutron and Muon Source (Rutherford Appleton Laboratory, UK). SANS profiles of the scattered neutron intensity were measured as a function of momentum transfer,

$$Q = \frac{4\pi \sin\theta}{\lambda}, \tag{S2}$$

where λ is the neutron wavelength and 2θ is the scattering angle. Spectra were collected using time-of-flight to determine the neutron wavelength, utilising a wavelength range of 0.9-13 Å. Two-dimensional scattering patterns were collected using a ^{3}He-tube array area detector (660 × 664 mm, 512 × 80 pixels) at a fixed position of 4.1 m from the sample. This instrument configuration covers an effective Q range of 0.004 - 0.67 Å^{-1}. SANS data were reduced using Mantid Workbench software.[3] Inspection of detector maps confirmed that all samples produced isotropic scattering enabling radial averaging of detector intensities after correcting for pixel efficiency. These data were normalised to the cumulative incident flux and divided by the

spectrum of the direct beam. SANS data obtained from polymer solutions were subtracted from SANS data corresponding to *n*-dodecane-$^{2}H_{26}$ collected under identical conditions. Finally, these data were placed on an absolute intensity scale using scattering from a standard sample (a solid blend of hydrogenous and deuterated polystyrene) in accordance with established procedures (Figure S2).

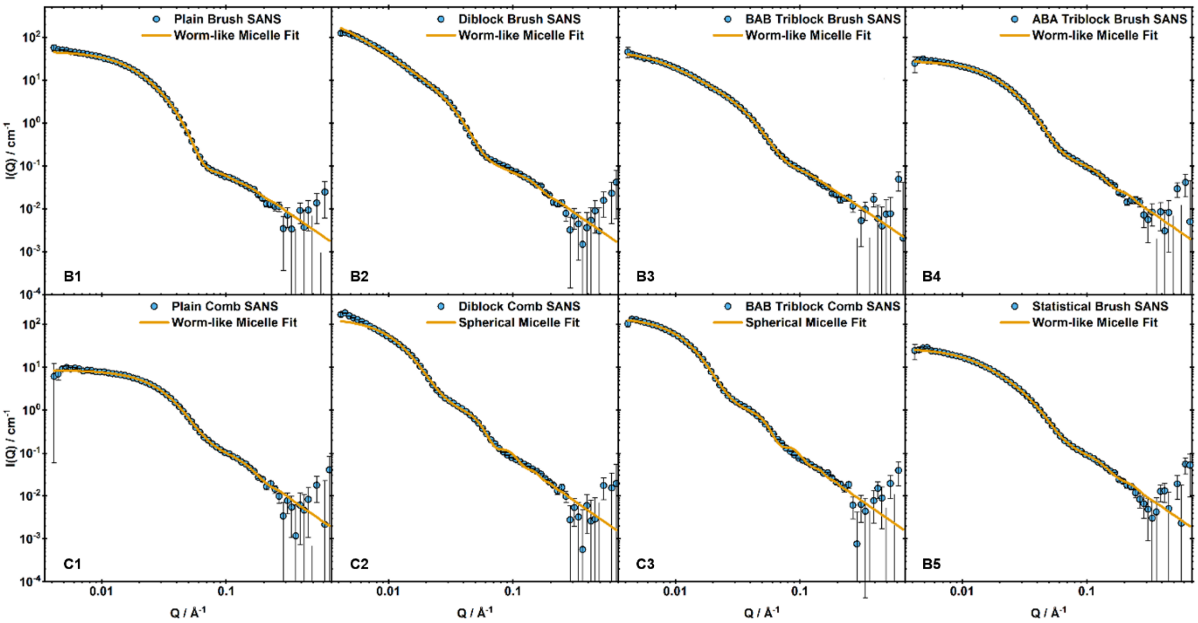

Figure S2. SANS profiles collected for 6 mg/mL bottlebrush and comb polymers in n-dodecane-$^{2}H_{26}$ (points) and corresponding fits (lines).

All SANS data were analysed using SASfit software.[4] Bottlebrush polymers B1-5 and comb C1 were fitted to a model describing a worm-like micelle (Table S1). The model describes a worm-like core (as described by Kholodenko[5]), where individually rigid segments with a length described by the Kuhn length are linearly connected and randomly oriented, over a total length described by the contour length. Each segment of the Kholodenko worm is then treated as a cylindrical micelle as described by Pedersen,[6] where a homogenous core is coated by polymer chains described by Gaussian coils. These assumptions were deemed suitable as PNAM segments would be expected to be contracted in a poor solvent such as *n*-dodecane, and PLA chains would be expected to behave as Gaussian coils due to their good solubility. In all cases, the core was assumed to have a scattering length density (SLD) of 1.24×10^{-6} Å^{-2}, corresponding to PHEAm/PNAM, and a shell SLD of 0.14×10^{-6} Å^{-2} corresponding to PLA.

In all cases, the volume of an individual graft was approximated to be 200,000 $Å^3$ assuming approximately DP 45 and that the volume of an individual LA repeat unit remains unchanged after polymerisation (calculated as 464 $Å^3$). The grafting density was fitted in all cases to account for the possibility of different backbone rigidity and compaction. Finally, we took the assumption that no solvent would be present in the core, due to the high targeted grafting density in the case of brush samples, and the relatively high proportion of polar moieties.

Combs C2 and C3 were fitted to a model describing a micelle with a spherical core grafted with Gaussian coil polymer chains (Table S1).[6] Broadly, the same constraints were applied as for the worm-like micelles described above. However, in this case, each "chain" in the corona would be expected to be a graft copolymer. We therefore allowed the volume of the "chains" in the corona to vary throughout the fitting procedure as we had no *a priori* information on their expected conformation in a micelle. The presence of worm-like structures in the corona was not accounted for explicitly in this model, meaning obtained structural parameters should be seen as an approximation of the self-assembled structures forming in solution.

Table S1. Parameters obtained through fitting SANS data of bottlebrush polymers to models describing worm-like or spherical micelles. Parameters marked by * were fixed throughout the fitting procedure.

Parameter	Bottlebrush polymer					Comb polymer		
	**B1**	**B2**	**B3**	**B4**	**B5**	**C1**	**C2**	**C3**
**Model**	Worm-like micelle	Worm-like micelle	Worm-like micelle	Worm-like micelle	Worm-like micelle	Worm-like micelle	Spherical micelle	Spherical micelle
**Scale**	0.028 ± 0.001	0.010 ± 0.002	0.073 ± 0.004	0.236 ± 0.004	0.186 ± 0.003	0.618 ± 0.008	0.117 ± 0.001	0.102 ± 0.001
**Core radius** (Å)	20.9 ± 0.034	19.5 ± 0.008	3.6 ± 0.0.014	22.6 ± 0.012	22.0 ± 0.007	17.3 ± 0.035	61.5 ± 0.001	64.2 ±
**Grafting density** ($Å^{-2}$)	0.002 ± 0.020	0.002 ± 0.007	0.009 ± 0.074	0.001 ± 0.002	0.001 ± 0.001	0.001 ± 0.002	0.001 ± 0.001	0.001 ± 0.001
**Brush Volume** ($Å^3$)	20000 *	20000 *	20000 *	20000 *	20000 *	20000*	111076 ± 0.004	100323 ± 0.005
**SLD Core** (10^{-6} $Å^{-2}$)	1.24*	1.24 *	1.24 *	1.24 *	1.24 *	1.24 *	1.24 *	1.24 *
**SLD Bush** (10^{-6} $Å^{-2}$)	0.14 *	0.14 *	0.14 *	0.14 *	0.14 *	0.14 *	0.14 *	0.14 *
**SLD Solvent** (10^{-6} $Å^{-2}$)	6.70 *	6.70 *	6.70 *	6.70 *	6.70 *	6.70 *	6.70 *	6.70 *
**Core Solvation** (%)	0 *	0 *	0 *	0 *	0 *	0 *	0 *	0 *
**Graft R_g** (Å)	17.8 ± 0.013	23.9 ± 0.003	22.1 ± 0.010	23.5 ± 0.003	21.4 ± 0.004	21.8 ± 0.023	83.4 ± 0.001	76.0 ± 0.001
**Kuhn Length** (Å)	64.5 ± 0.017	251.3 ± 0.005	325.0 ± 0.042	178.3 ± 0.070	311.6 ± 0.073	57.8 ± 0.095	-	-
**Contour Length** (Å)	815.3 ± 0.018	4980.8 ± 0.692	934.5 ± 0.009	344.0 ± 0.011	471.4 ± 0.005	201.8 ± 0.022	-	-
**d**[A]	-	-	-	-	-	-	0.77 ± 0.009	0.87 ± 0.011

[A] Parameter d is a measure of chain interpenetration into the spherical core of the micelle. For non-penetrating chains d ≈ 1, whereas for brush penetration into the core d > 1.

2.7 Polarised Neutron Reflectometry (PNR)

Neutron reflectometry, including the application of polarisation for structural analysis of soft matter interfaces, has been described in detail elsewhere,[7-10] so here we include only a brief description of the technique to aid in interpretation of our results. Neutron reflectometry measures the elastic specular reflected intensity of a collimated neutron beam from an interface as a function of the momentum transfer vector perpendicular to the interface, Q_z, defined by

$$Q_z = \frac{4\pi sin\theta}{\lambda}, \tag{S3}$$

where θ is the incident angle and λ is the incident neutron wavelength. At any given value of Q_z, the reflected intensity is dependent on the structure and SLD of material adsorbed to the interface and surrounding bulk material, as neutrons are partially reflected and refracted at each interface encountered along the path length. The SLD, ρ, of a molecule is defined as the sum of coherent atomic scattering lengths (b_c) for each of N nuclei within a molecule of a given molecular volume, V_m. For polymeric materials, definition of a precise molecular volume can be challenging, therefore it can also be described in terms of mass density, ρ_m, molecular weight, M_w, and Avogadro's constant, N_A, as used throughout this work:

$$\rho = \frac{\sum_{i=1}^{N} b_c}{V_m} = \frac{\rho_m N_A \sum_{i=1}^{N} b_c}{\sum_{i=1}^{N} M_w}. \tag{S4}$$

Notably for this study, neutrons are highly sensitive to differences in SLD to hydrogenated and deuterated materials, allowing differential isotopic labelling of bulk solvent and adsorbed organic films to dramatically increase the achievable contrast between chemically similar phases. As a result of their non-integer spin, neutrons are also spin polarisable. When interacting with magnetic material in a magnetic field, polarised neutrons effectively experience different SLDs depending on their spin state. A combination of magnetic reference layers within the substrate and isotopic labelling of the bulk solvent allows exploitation of magnetic and isotopic contrast variation. This approach has been shown to be capable of resolving complex chemical interfaces with low ambiguity.[9-11]

Si-Py-Steel substrates were cleaned by UV-ozone irradiation for 20 min and extensively washed with ultrapure water. Substrates were then subject to successive sonication in aqueous 2 w/v% SDS, EtOH and toluene. After thoroughly drying under a stream of nitrogen gas, substrates were ozone cleaned, rinsed thoroughly with EtOH and mounted into PEEK laminar flow cells submerged in ethanol in order to avoid introduction of air bubbles during cell assembly.

Polarised neutron reflectometry was performed on the POLREF reflectometer at the ISIS pulsed neutron and muon source (UK) operating in polarised mode using fast flipping such that the neutron spin state is reversed with every neutron pulse, giving ≥98% polarisation. Flow cells were mounted in horizontal geometry, connected to a glass syringe containing either

hydrogenated or deuterated dodecane via PTFE tubing mounted in a syringe pump, calibrated and set to a flow rate of 0.5 ml/min throughout. Aluminium top plates of the flow cells were connected to a water bath and the temperature maintained at 45 °C. Iron yokes were placed over the flow cells to ensure a constant magnetic field experienced throughout the sample. PNR was measured at 0.5°, 1.5° and 2.5° incident angles using an incident wavelength range of 2-15 Å covering an effective Q_z range of 0.01-0.3 $Å^{-1}$, where $\delta Q/Q$ is 3%. Inefficiencies in neutron polarisation were corrected for as described by Wildes *et al*. The resultant reflectivity patterns were normalised to the incident flux measured in transmission through each substrate.

The substrate was initially characterised by PNR in two isotopic contrasts, hydrogenated and deuterated *n*-dodecane, with spin up (↑) and spin down (↓) polarised neutrons, yielding four reflectivity curves describing the same interfacial structure. Sample solution (0.1 % w/v in *n*-dodecane) was injected into the flow cell *via* the syringe pump at a concentration of and incubated for 2 h. The cell was then flushed with hydrogenated *n*-dodecane to remove polymer remaining in the bulk phase and data was collected with both magnetic contrasts, after which deuterated *n*-dodecane was injected into the cell to repeat the measurements.

Data were analysed using RasCAL.[6] This software calculates a reflectivity pattern resulting from a theoretical SLD profile consisting of two bulk phases with a series of interfacial layers each treated as homogeneous ‘slabs’. Each layer within this model is defined by four parameters: thickness, SLD, roughness, and solvation. The four datasets corresponding to the bare substrate were fitted to include four layers, corresponding to SiO_2, permalloy, steel and a thin oxide layer on the surface. These four datasets were co-refined using *a priori* information, such as the SLD of silicon and SiO_2, to obtain a structural model corresponding to the substrate. The parameters associated with this model were then fixed in the analysis of the polymer layer. To fit the data acquired with the adsorbed polymer layer, two additional layers were required, corresponding to the linear PNAM and the grafted PLA segments.

The error associated with the parameters in the model were estimated by Markov chain Monte Carlo (MCMC) analysis, enabling covariance between parameters to be accounted for. Here, a Gaussian prior distribution was assumed for all parameters. The posterior distribution was determined by performing 5,000 burn-in iterations for location of the global minima prior to a further 50,000 iterations used to define the posterior distribution. Three independent

repeats of this analysis were used to calculate the asymmetric 95% confidence intervals associated with the fits and model parameters.

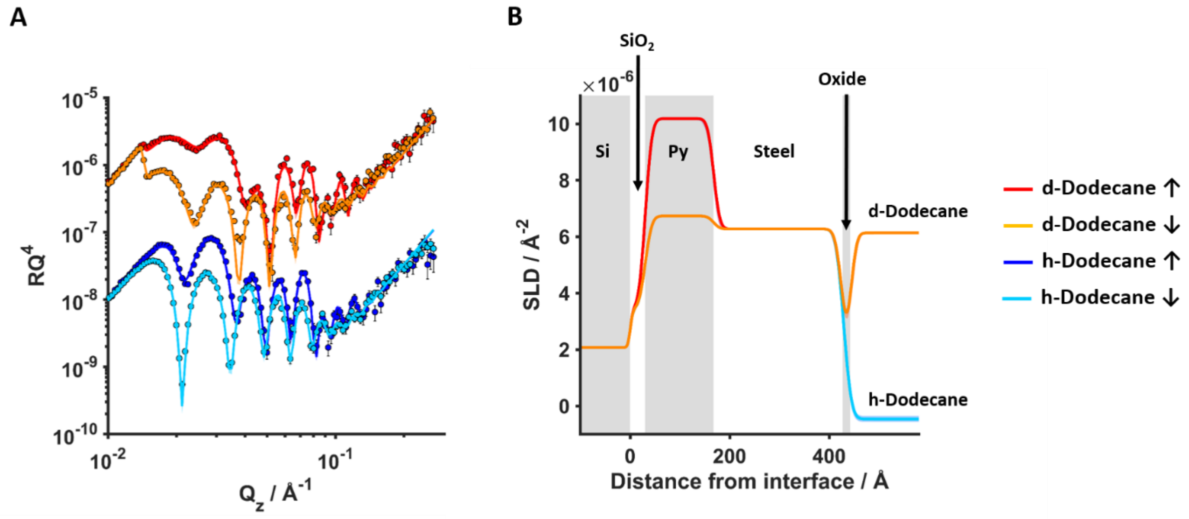

Figure S3. A) Polarised neutron reflectometry data (points) and fits (lines), plotted as RQ^4, corresponding to Si-Py-Steel substrates characterised in h- and d-dodecane isotopic contrasts with spin up (↑) and spin down (↓) magnetic contrasts. For clarity, data and fits corresponding to d-Dodecane isotopic contrasts have been vertically offset. **B**) SLD profiles corresponding to fits shown in a. Grey shaded regions indicate discrete layers included in the model. Throughout, colored shaded regions indicate the 95% confidence interval associated with the fit/ model as determined by MCMC.

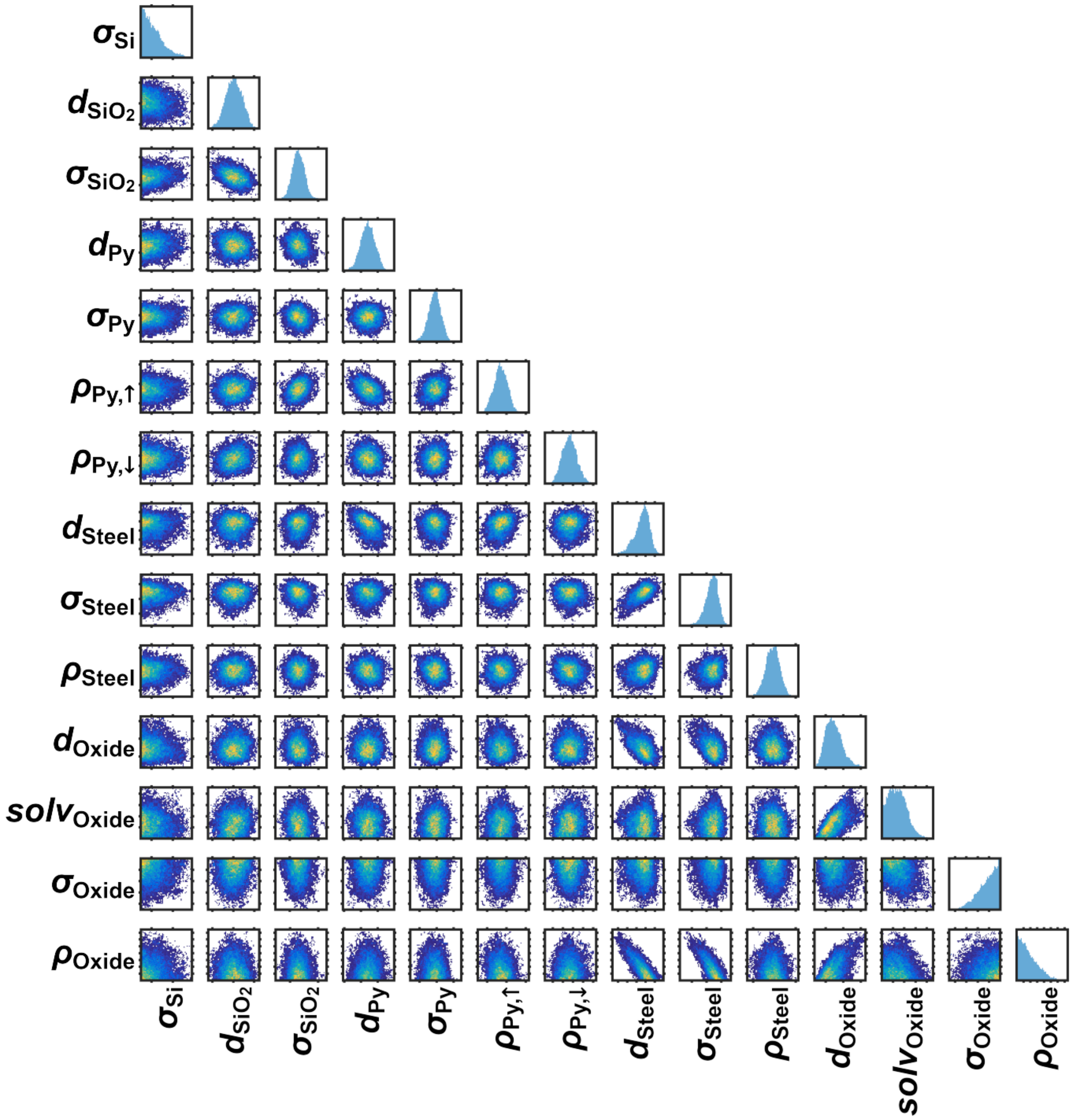

Figure S4. Correlation plot for fits to PNR data for Si-Py-Steel substrates prior to polymer incubation. Histograms show the posterior distribution associated with each parameter as determined by MCMC analysis. Heatmaps show parameter pairs plotted against another for each of the 50,000 MCMC iterations to show covariance between parameters, where layer thickness is represented by ***d***, roughness by ***σ***, SLD by ***ρ*** and solvation by ***solv***. Subscript notations indicate the layer with which the parameter is associated.

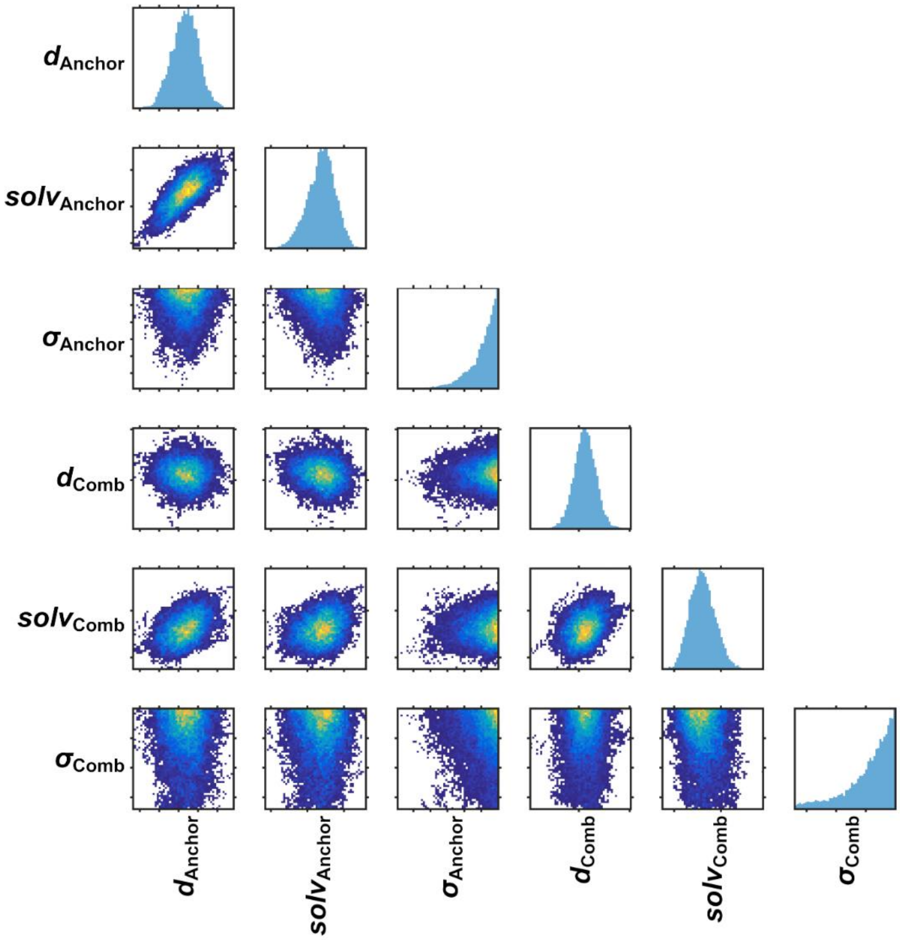

Figure S5. Correlation plot for fits to PNR data for Si-Py-Steel substrates after polymer incubation. Histograms show the posterior distribution associated with each parameter as determined by MCMC analysis. Heatmaps show parameter pairs plotted against another for each of the 50,000 MCMC iterations to show covariance between parameters, where layer thickness is represented by $\boldsymbol{d}$, roughness by $\boldsymbol{\sigma}$, SLD by $\boldsymbol{\rho}$ and solvation by $\boldsymbol{solv}$. Subscript notations indicate the layer with which the parameter is associated.

2.8 Viscosity Measurements

Viscosity measurements were carried out on a ISL Houillon viscometer according to ASTM D7279 (Standard Test Method for Kinematic Viscosity of Transparent and Opaque Liquids by Automated Houillon Viscometer).

Table S2: Viscosity of polymer oil blends at 1 wt.% measured at 40 and 100°C.

	**Base oil**	**PLA Brush** (B1)	**Diblock** (B2)	**BAB** (B3)	**ABA** (B4)	**Statistical** (B5)	**PMA**
**Viscosity @40 cSt**	19.23	26.27	20.43	20.33	20.11	20.54	20.05
**Viscosity @100 cSt**	4.25	5.4	4.47	4.46	4.4	4.5	4.39
**VI**	129	146	134	133	132	136	132

3 Synthetic protocols

3.1 Synthesis of di-BIBDTC

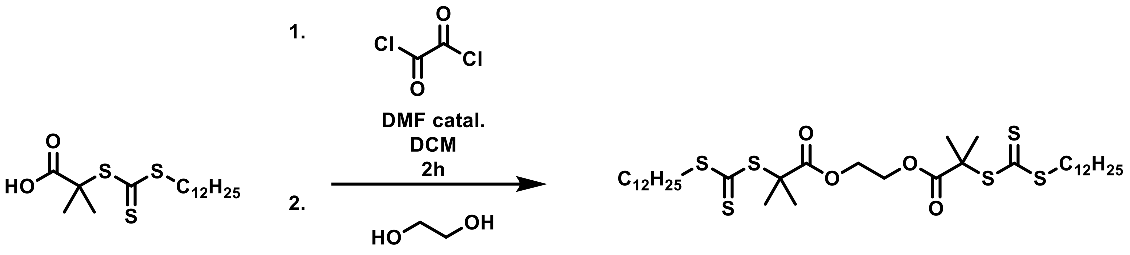

Scheme S1. Synthesis of difunctional RAFT agent di-BIBDTC.

Dry glassware and anhydrous solvents were used for the reaction. IBADTC (1.51 g, 4.1 mmol, 3 eq.) was dissolved in DCM in a round-bottom flask. 2 M oxalyl chloride solution (4.1 ml, 8.2 mmol, 6 eq.) was added under vigorous stirring and nitrogen flow. Reaction was started by adding a drop of DMF ($CO\uparrow$, $CO_2\uparrow$). Stirring was continued for 2 h, after which DCM and excess oxalyl chloride were removed using a Schlenk line. The oil was re-dissolved in DCM. In a separate flask, ethylene glycol (85 mg, 1.4 mmol, 1 eq.) was weighed and dissolved in DCM. After setting the flask on ice, acyl chloride solution was added dropwise under nitrogen flow. The flask was taken off ice and nitrogen flow was removed after the reaction mixture had reached room temperature. Stirring was continued for 12 h.

The solution was diluted and washed thrice with 1.0 M $NaHCO_3$ and twice with brine. The organic phase was dried over $MgSO_4$ overnight, filtered, and DCM was removed using a rotary evaporator. A flash column was used to isolate the pure CTA by running an elution gradient of pure hexane to 5% ethyl acetate. Solvents were removed and the product was dried in a vacuum oven at 40 ^{o}C overnight. Storing the product in the fridge gave a yellow crystalline product (0.89 g, 86%).

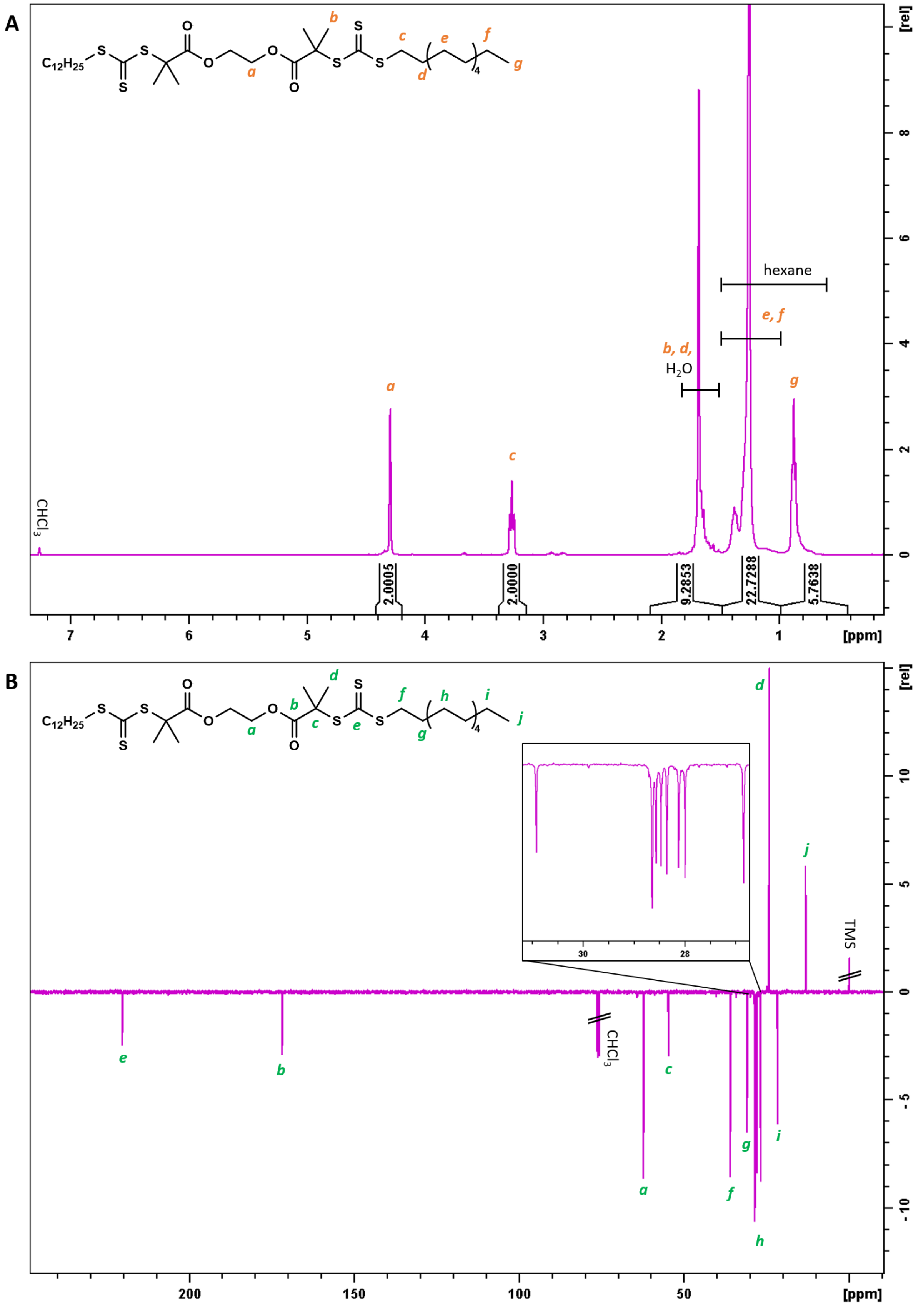

Figure S6. ^{1}H NMR (**A**) and ^{13}C NMR (**B**) analysis of di-BIBDTC in $CDCl_3$.

3.2 Polymerisation of backbones for the mono- and diblock structures

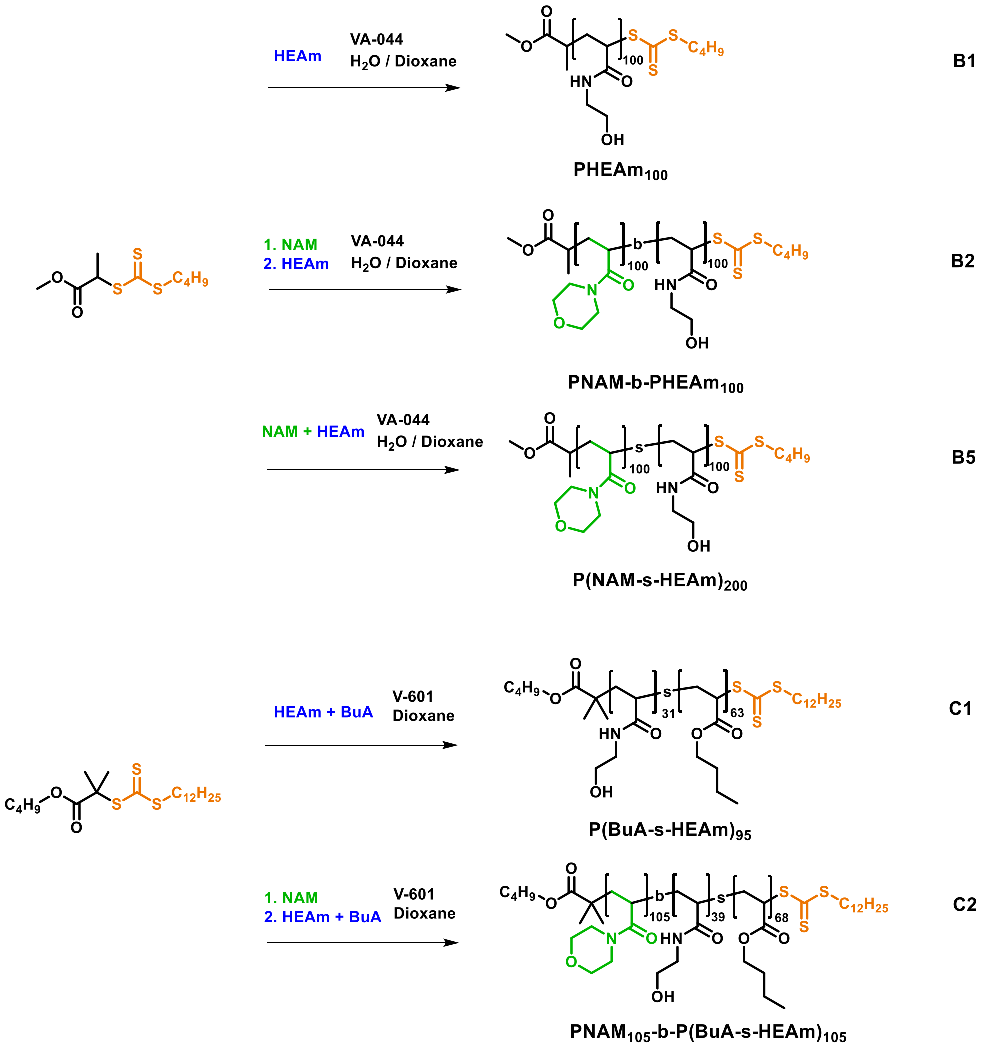

Scheme S2. Synthetic routes used for polymerising the backbones of brush (B1), diblock brush (B2), statistical brush (B5), comb (C1) and diblock comb (C2) copolymers.

3.2.1 Backbone synthesis for densely grafted brushes B1, B2 and B5

NAM (1g, 7.08 mmol), PMBTC (17.9 mg, $7.08*10^{-2}$ mmol), VA-044 (0.6 mg, $1.77*10^{-3}$ mmol) were dissolved in a mixture water (1590 µl) and dioxane (1060 µl) in a 7 ml vial fitted with a stirrer bar and rubber septum. The reaction mixture was degassed with N_2 for 15 minutes and placed in an oil bath heated to 44 °C for 7 h. A sample was taken to confirm full consumption of monomer by NMR and the vial stored in the fridge overnight. Then a degassed of HEAm (815 mg, 7.08 mmol), VA-044 (0.6 mg, $1.77*10^{-3}$ mmol) and water (300 µl) was added to the vial, mixed and heated at placed in an oil bath heated to 44 °C for 7 h. NMR showed 94% monomer conversion, the reaction mixture was diluted with methanol and

precipitated once in acetone, twice into ethyl acetate and dried in a vacuum oven at 40 °C overnight to yield a pale yellow powder.

For the $PHEAm_{100}$ homopolymer backbone the same reaction conditions as for the first block were used but with HEAm (7.08 mmol) instead of NAM. For the statistical $PNAM_{100}$-co-$PHEAm_{100}$ backbone a polymerisation mixture of NAM (920 mg, 6.52 mmol), HEAm (750 mg, 6.52 mmol), PMBTC (16.4 mg, $6.52*10^{-2}$ mmol), VA-044 (0.5 mg, $1.77*10^{-3}$ mmol), water (2115 µl) and dioxane (1410 µl) was prepared and reacted in the same conditions as described above.

3.2.2 Backbone synthesis for loosely grafted combs C1 and C2

For preparing the statistical P(HEAm-*s*-BuA)$_{95,35\%}$ copolymer backbone, BuA (1.00 g, 7.80 mmol), HEAm (0.360 g, 3.13 mmol), BIBDTC (32.7 mg, $7.77*10^{-2}$ mmol) and V-601 (1.30 mg, $5.65*10^{-3}$ mmol) were dissolved in dioxane (3300 µl) in a 7 ml vial fitted with a stirrer bar and rubber septum. The reaction mixture was degassed with N_2 for 15 min and placed in an oil bath heated to 70 °C. Samples were taken to monitor the consumption of monomers by ^{1}H NMR and the reaction was stopped after 1.5 h at 62% and 76% conversion of BuA and HEAm, respectively. The reaction mixture was diluted and the polymer was precipitated thrice into methanol/water mixture (4:1) and dried in vacuum oven at 40 °C.

For preparing the diblock copolymer $PNAM_{105}$-*b*-P(HEAm-*s*-BuA)$_{105,35\%}$, NAM (1.00g, 7.08 mmol), BIBDTC (23.0 mg, $5.47*10^{-2}$ mmol), V-601 (0.63 mg, $2.73*10^{-3}$ mmol) were first dissolved in dioxane (1470 µl) and polymerised using the protocol described above. Reaction was stopped after 30 min at 77% conversion and the polymer was precipitated three times into diethyl ether and dried yielding a pale yellow powder. The polymer (0.530 g, $3.51*10^{-2}$ mmol) was then dissolved in dioxane (1.83 ml) along with BuA (467 mg, 3.64 mmol), HEAm (171 mg, 1.49 mmol) and V-601 (0.57 mg, $2.48*10^{-3}$ mmol). Polymerisation was carried out at 70°C for 1.5 h and reaction was stopped at 65% and 91% conversion for BuA and HEAm, respectively. The polymer was purified by precipitation into cold diethyl ether and dried under vacuum.

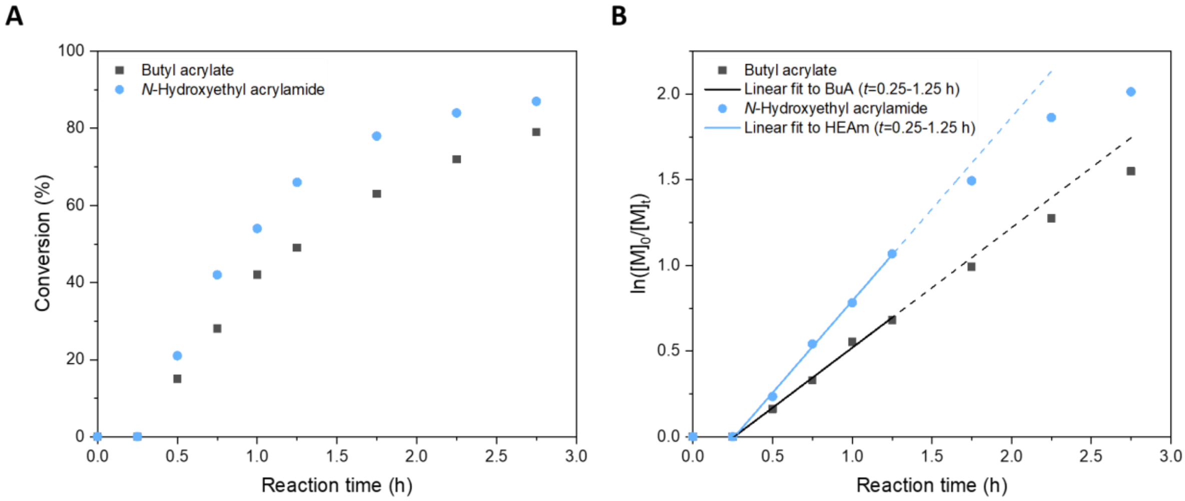

Figure S7. Kinetic data for the copolymerisation of butyl acrylate and *N*-hydroxyethyl acrylamide. Conversion data (A) was converted into a pseudo first-order linear plot (B). Linear fits (t = 0.25-1.25 h, $r^2 \geq 0.995$) and their interpolations are indicated by solid and dashed lines, respectively.

3.3 Polymerisation of backbones for the triblock structures

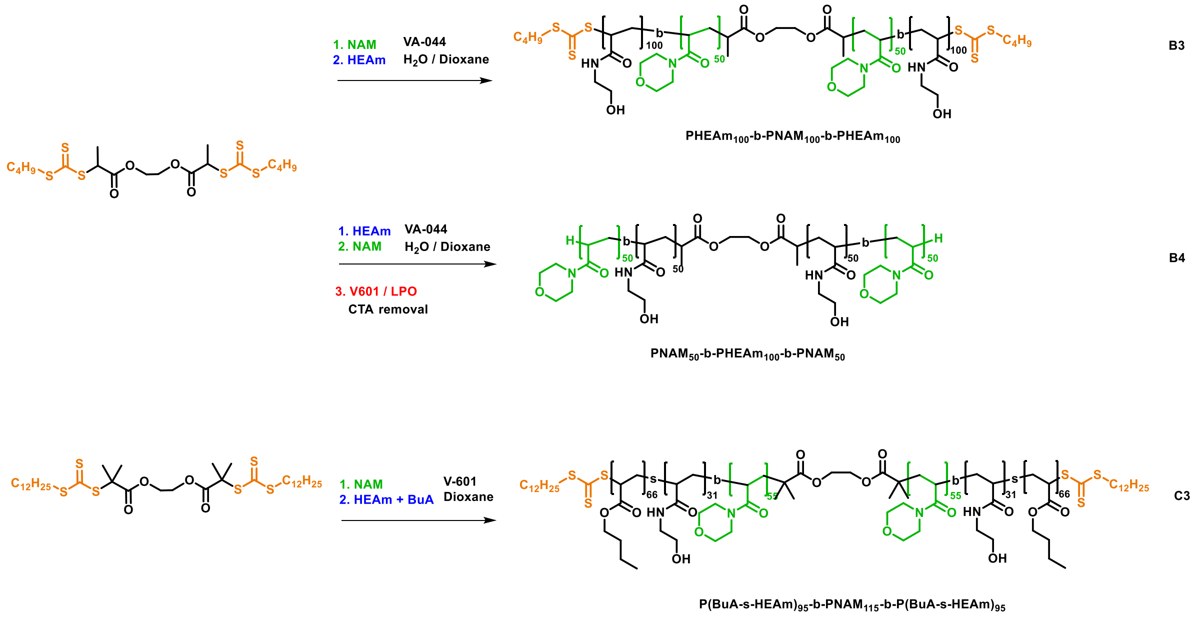

Scheme S3: Synthetic routes used in the polymerisation of triblock B3, B4and C3 backbone copolymers.

3.3.1 B*ackbone **synthesis*** for densely grafted brush ***B4***

HEAm (750 mg, 6.51 mmol), DiPABTC (32.8 mg, $6.51*10^{-2}$ mmol), VA-044 (0.7 mg, $2.17*10^{-3}$ mmol), water (397 µl) and dioxane (748 µl) were placed in a 3 ml vial fitted with a

stirrer bar and rubber septum. The reaction mixture was degassed with N_2 for 15 minutes and placed in an oil bath heated to 44°C for 8h. NMR showed 97 % conversion of monomer, the reaction mixture was precipitated twice into acetone and dried under vacuum at 40°C. 400 mg of the dried product was then mixed with NAM (490 mg, 3.47 mmol), VA-044 (0.4 mg, $1.16*10^{-3}$ mmol) and water (1300 µl), degassed with N_2 and heated at 44°C for 6h. The reaction mixture was precipitated into acetone and then twice in ethyl acetate and dried under vacuum.

A previously reported procedure was adapted for the CTA end group removal.[12] The ABA $PNAM_{50}$-b-$PHEAm_{100}$-$PNAM_{50}$ polymer (1 g, 1 eq. with respect to CTA), AIBN (230 mg, 20 eq.) and lauroyl peroxide (40 mg, 2 eq.) were dissolved in 15 ml DMF, degassed for 10 minutes with N_2 and placed in an oil bath heated to 80°C for 6h. The reaction mixture was precipitated three times into diethyl ether and dried under vacuum to yield a colourless powder. Analysis by SEC with a UV detector showed no absorption from the polymer at 309nm indicating quantitative removal of the CTA end group.

3.3.2 ***B**ackbone **synthesis** for densely grafted brush **B3***

Synthesis was performed without precipitation between blocks using the same procedure as above except addition of the NAM block first and the HEAm second.

3.3.3 ***B**ackbone **synthesis** for loosely grafted comb **C3***

Synthesis was carried out by using the same protocol and reaction conditions as for diblock comb but using the difunctional CTA di-BIBDTC. In the first step $[M]_0 = 3$ M, $[NAM]_0/[CTA]_0 = 130$ and $[CTA]_0/[I]_0 = 20$. In the second step $[M]_0 = 2$ M, $[BuA]_0/[HEAm]_0/[mCTA]_0 = 200/80/1$ and $[CTA]_0/[I]_0 = 14$.

3.4 Functionalisation of backbones

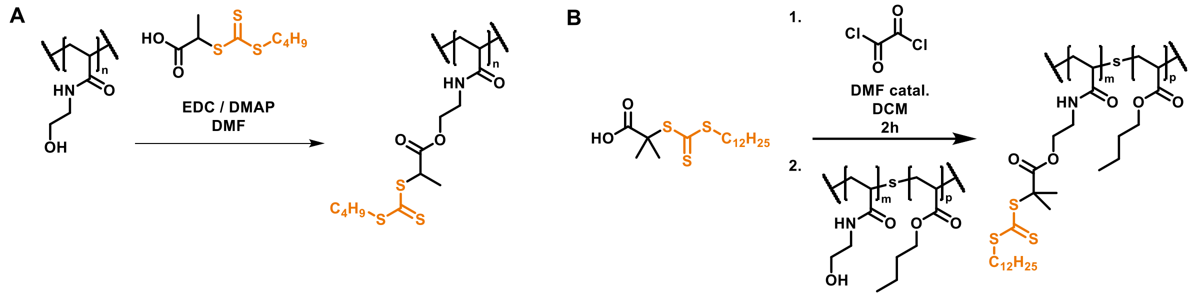

Scheme S4. General synthetic routes used in the functionalisation of brush backbones (**A**) and comb backbones (**B**) with CTAs.

3.4.1 Densely grafted brushes

PHEAm copolymer (500 mg, 1 eq. with respect to alcohol groups) was dissolved in 10 ml anhydrous DMF under nitrogen and PABTC (1.5 eq.) added. The reaction mixture was cooled with an ice bath followed by addition of DMAP (0.15 eq.) then EDC (2 eq.). After two hours the ice bath was removed and left to stir overnight. The reaction mixture was concentrated under a stream of nitrogen and precipitated three times into an ice cold solution of methanol / water (50:50), solubilising in dioxane between precipitations. The precipitate was transferred to a vial and dried under vacuum at 40°C to yield a sticky orange solid.

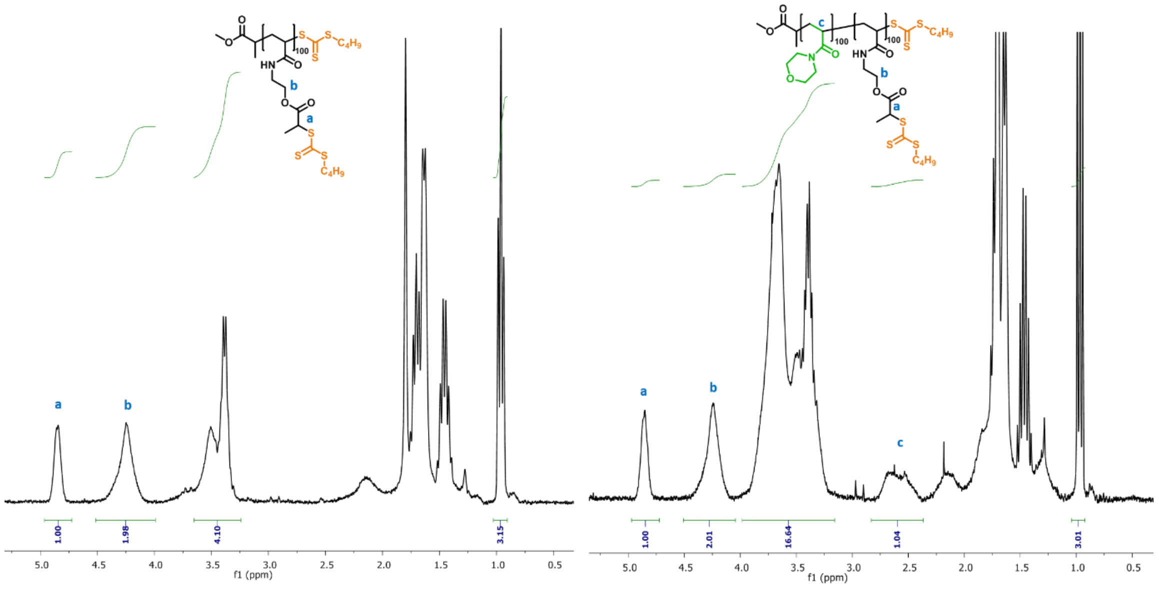

Figure S8. Left: ^{1}H NMR spectra in $CDCl_3$ of PCTA derived from homopolymer $PHEAm_{100}$ backbone. **Right**: ^{1}H NMR spectra in $CDCl_3$ of diblock PCTA backbone showing integration of 'a' CH adjacent to trithiocarbonate has approximately the same integral as environment 'c' which is consistent with an equal DP 100 for the PNAM and PCTA blocks.

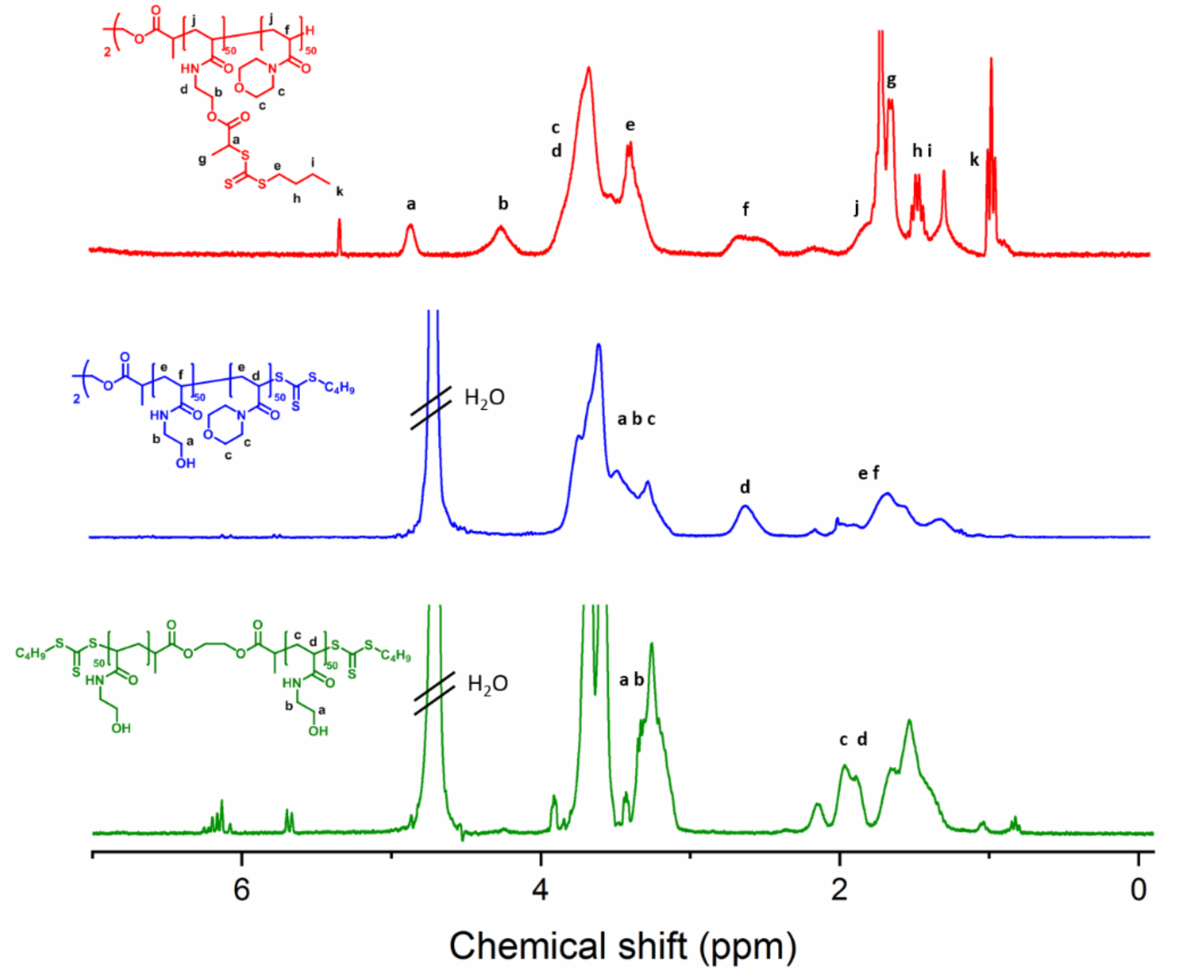

Figure S9. ^{1}H NMR analysis of polymerisation mixtures. **Bottom**: PHEAm middle block in D_2O. Monomer conversion was determined to be 95% and the polymer was purified by precipitation prior to chain extension. **Middle**: B4 triblock copolymer backbone in D_2O after chain-extension of PHEAm with NAM. **Top**: PolyCTA, obtained from the functionalisation of the B4 triblock copolymer backbone, in $CDCl_3$.

3.4.2 Loosely grafted combs

In a dry round-bottom flask equipped with a stir bar, P(BuA-*s*-HEAm) was dissolved in anhydrous DCM. In a separate flask, IBADTC (2 eq. with respect to HEAm units) was dissolved in anhydrous DCM and oxalyl chloride solution (4 eq.) was added to the solution under nitrogen flow and vigorous stirring. A catalytic amount of anhydrous DMF was added to start the reaction ($CO\uparrow$, $CO_2\uparrow$) and stirring was continued for 2 h, after which DCM and excess oxalyl chloride were removed using a Schlenk line. The red oil was redissolved in DCM and added dropwise to P(BuA-s-HEAm) solution set in an ice bath. After two hours the ice bath was removed and left to stir overnight. The reaction mixture was concentrated by rotary

evaporation and precipitated into methanol until no free CTA remained in the product ($n \geq 3$). Solvent residue was removed by rotary evaporation and drying in a vacuum oven at 40°C.

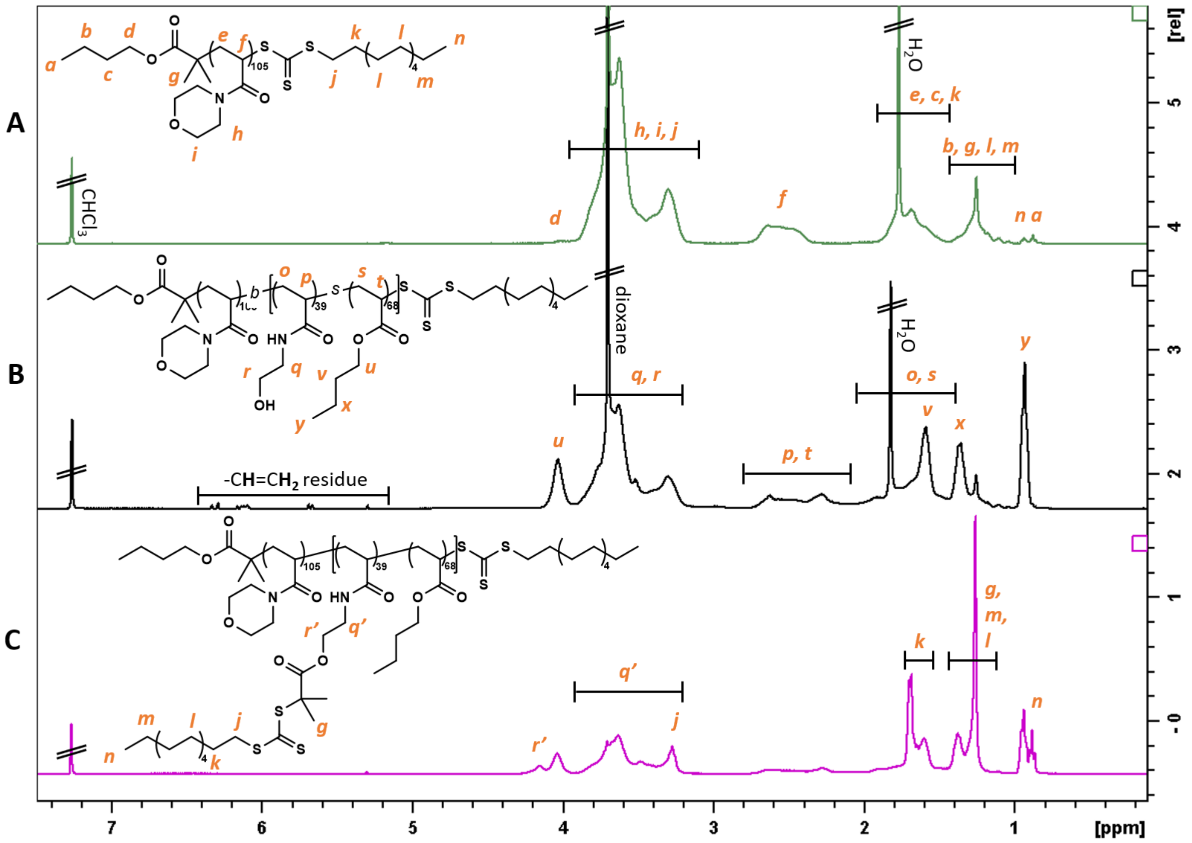

Figure S10. ^{1}H NMR spectra of linear PNAM (**A**) chain-extended with BuA and HEAm (**B**) and further functionalised with CTA acid to yield a polyCTA (**C**). Sample solutions were prepared in $CDCl_3$.

3.5 Polymerisation of PLA side chains

3.5.1 Densely grafted brushes

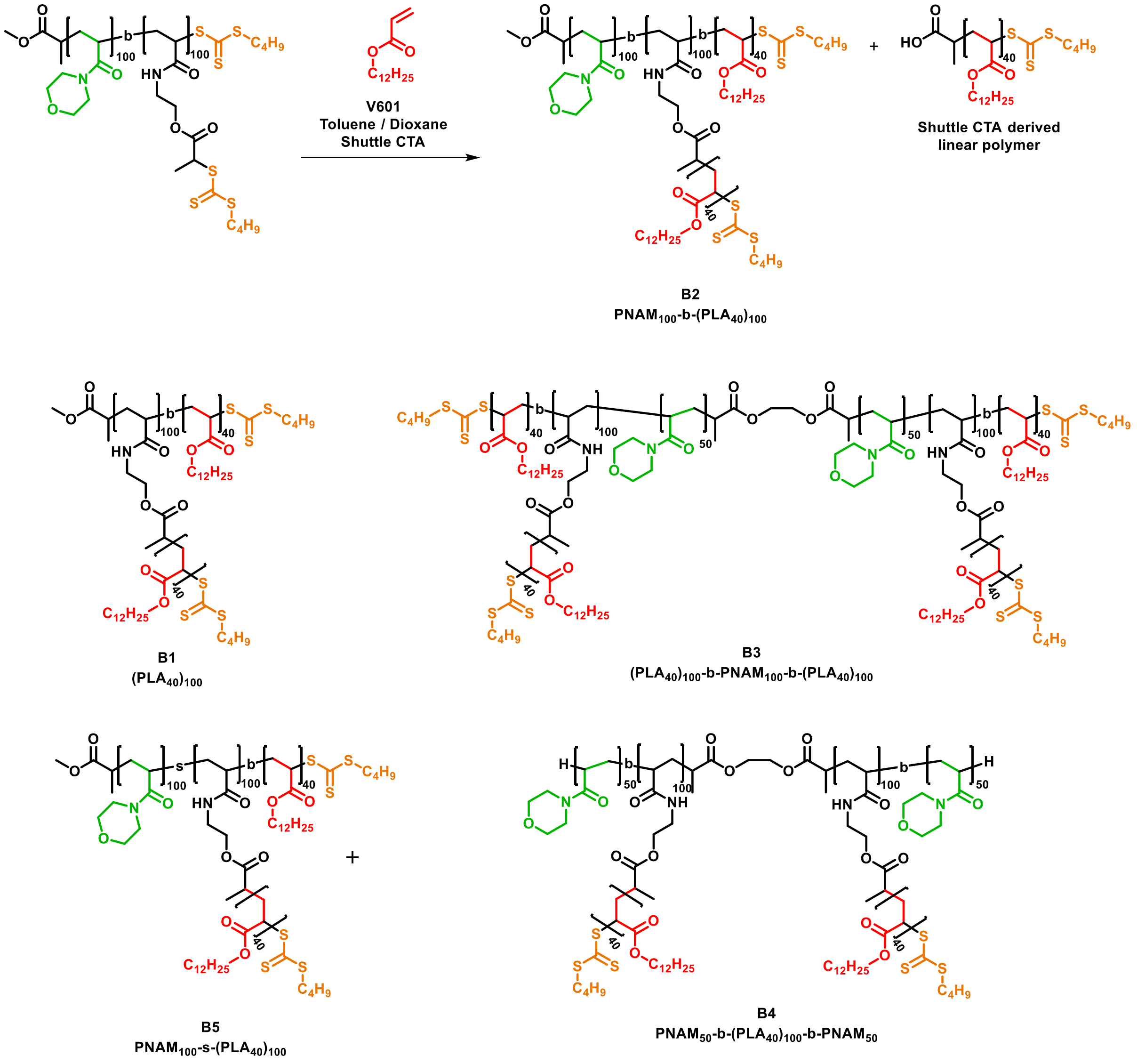

Scheme S5. General synthetic route used for the R-group *grafting from* polymerisation of densely grafted brushes using a shuttle CTA and resulting structures. All products contained a by-product of linear PLA.

A DP of 75 was targeted for the side chains with the addition of 0.4 eq. free shuttle CTA with respect to the polymeric grafted CTA units. Lauryl acrylate (8g, 33.28 mmol), PolyCTA (0.317 mmol), PABTC (0.127 mmol), V601 (3.8 mg, $1.66*10^{-2}$ mmol), toluene (12.1 ml) and dioxane (12.1 ml) were placed in 50 ml round bottom flask. For the homopolymer $PCTA_{100}$ backbone the polymerisation was performed in 100% toluene as the solvent at the same overall concentration. The reaction mixture was degassed with nitrogen and placed in an

oil bath set to 65C for 7.5 hours, until a monomer conversion of 55 – 60 % was obtained to reach a side chain length of DP 40-45. The flask was cooled to room temperature and the reaction mixture precipitated three times into ice cold methanol and dried under vacuum to yield yellow oil.

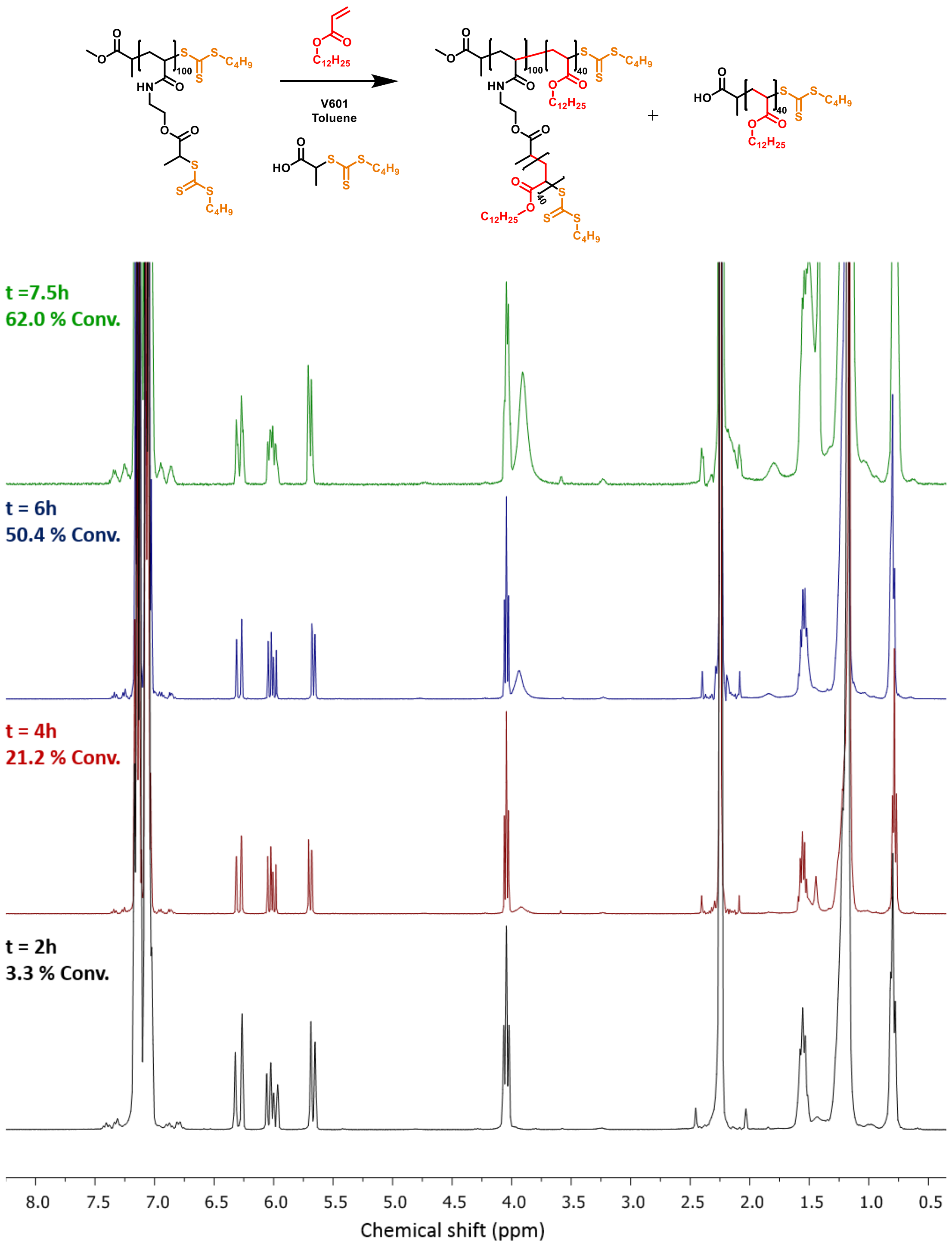

Figure S11. ^{1}H NMR spectra in $CDCl_3$ showing grafting from kinetics of the DP 100 pCTA backbone. Monomer conversions were determined by integration of the CH_2 ester peak of monomer + polymer at 3.8-4.1 ppm against monomer vinyl peaks at 6 ppm.

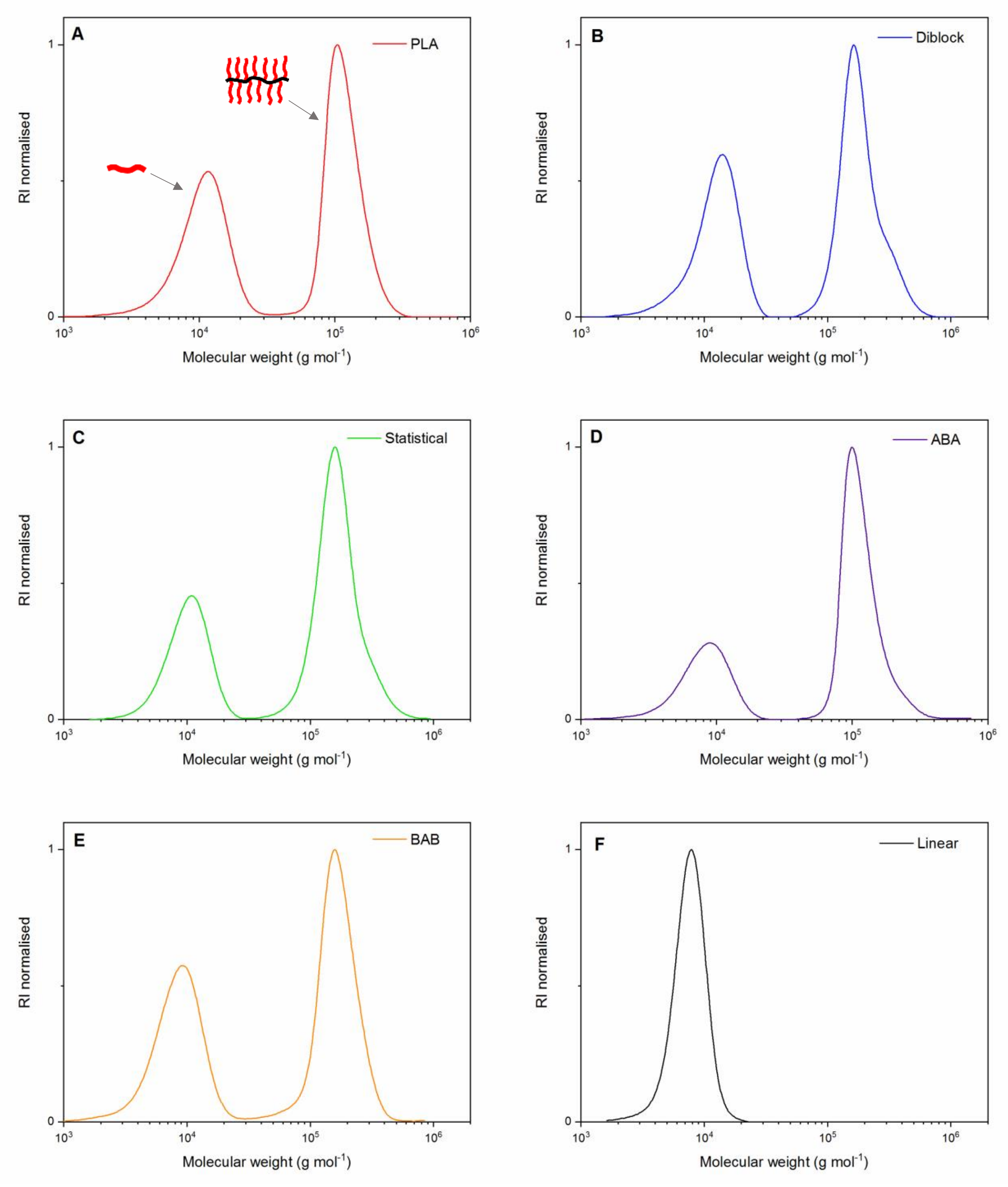

Figure S12. SEC chromatograms of bottlebrush samples including linear polymer trace at low molecular weight. The wt. % fraction of linear polymer species was calculated by comparing the relative areas of the RI signals for the brush/linear species. PLA brush – 42 % linear, Diblock – 42 %, Statistical – 33 %, ABA – 30 %, BAB – 42 %.

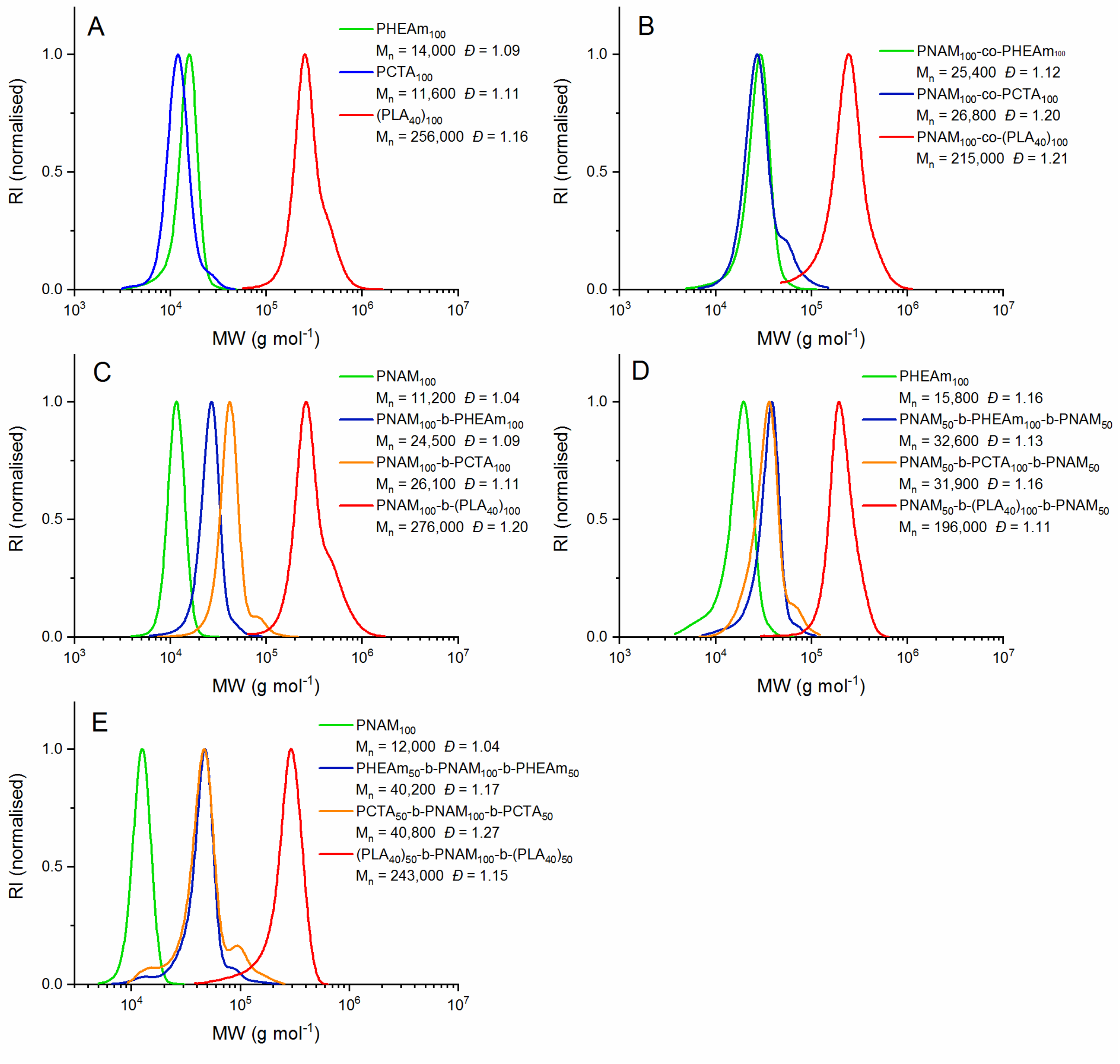

Figure S13. SEC chromatograms of **A**) PLA brush B1, **B**) statistical brush B5, **C**) diblock brush B2, **D**) ABA triblock brush B4, and **E**) BAB triblock brush B3. The backbones and polyCTA polymers were analysed with DMF SEC, whereas the final PLA bottlebrushes were analysed with $CHCl_3$ SEC due to differences in solubility. The PLA bottlebrushes contain shuttle CTA derived linear polymer chains which have been omitted for clarity, but are shown above in Figure S12.

3.5.2 *Loosely grafted combs*

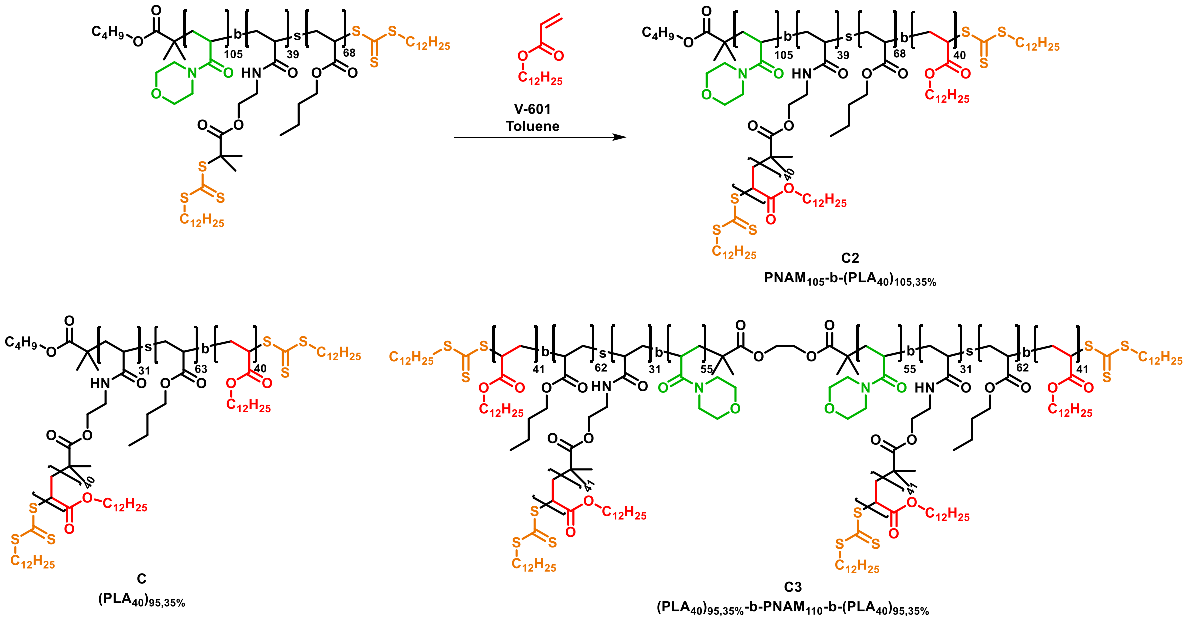

Scheme S6. General synthetic route used for the R-group *grafting from* polymerisation of loosely grafted combs.

A DP of 55 was targeted for the side chains; no shuttle CTA was used. Lauryl acrylate, polyCTA, V-601 ($[CTA]_0/[I]_0 = 25$) and toluene ($[M]_0 = 1$ M) were placed in a round bottom flask with a stirrer. The reaction mixture was degassed with nitrogen and placed in an oil bath set to 70 ^{o}C for 5.5 hours, until a monomer conversion of 71-76 % was obtained to reach a side chain length of DP 39 – 42. The flask was cooled to room temperature and the reaction mixture precipitated three times into cold methanol and dried under vacuum.

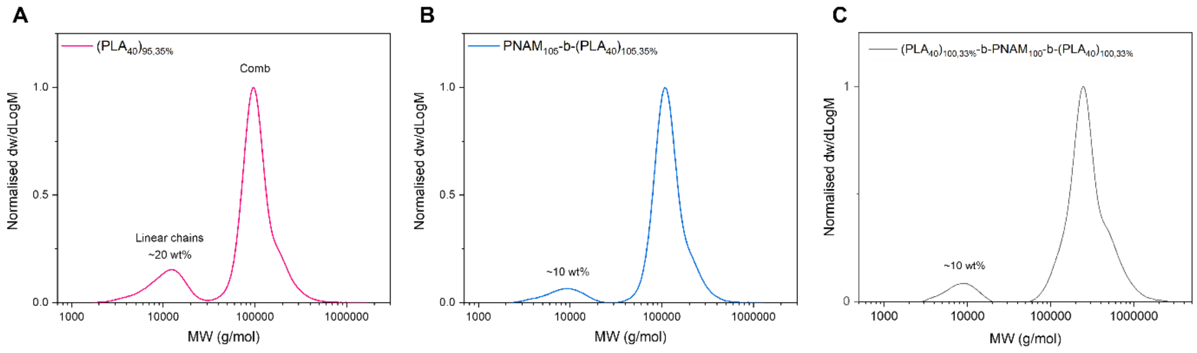

Figure S14. Size-exclusion chromatograms of the final product and the linear by-product for **A**) PLA comb C1, **B**) diblock comb C2 and **C**) BAB triblock comb C3.

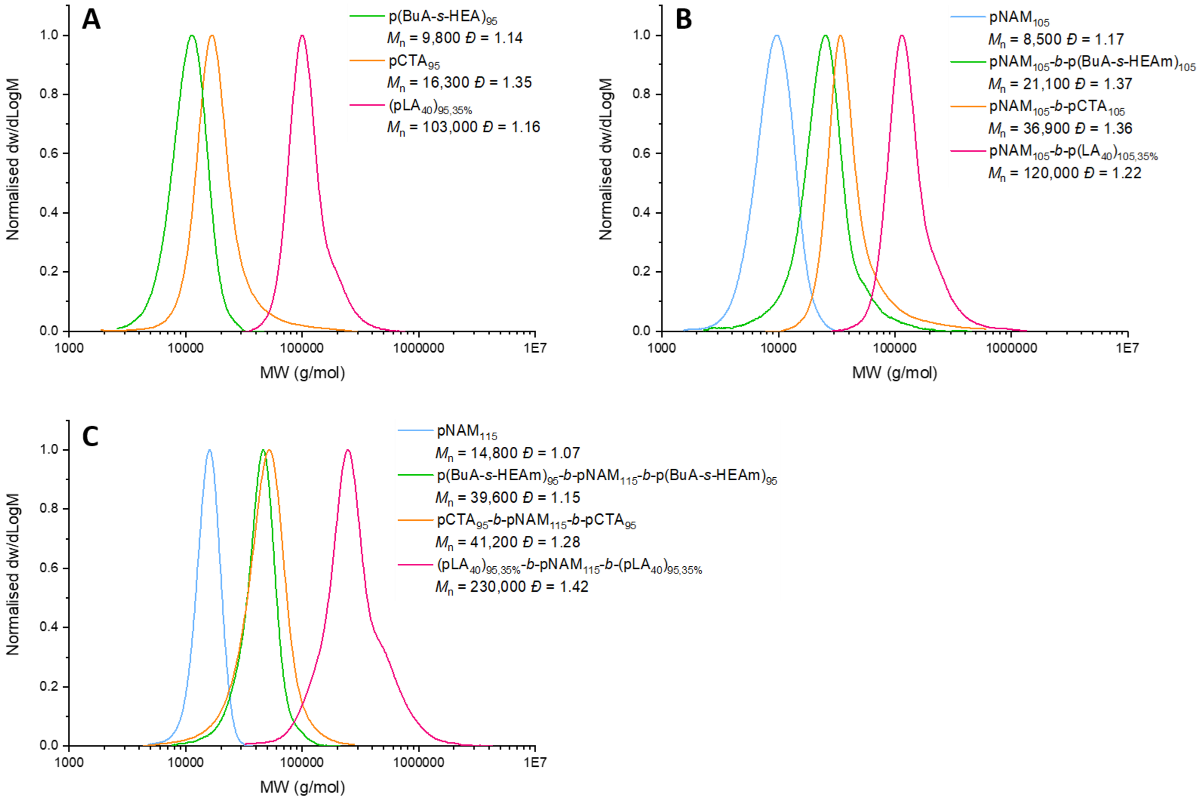

Figure S15. Size-exclusion chromatograms of each intermediate and the final product in the synthesis of **A**) PLA comb C1, **B**) diblock comb C2, and **C**) BAB triblock comb C3. The backbones, polyCTA polymers and the final comb polymers were analysed with DMF SEC, $CHCl_3$ SEC and THF SEC, respectively, due to differences in solubility.

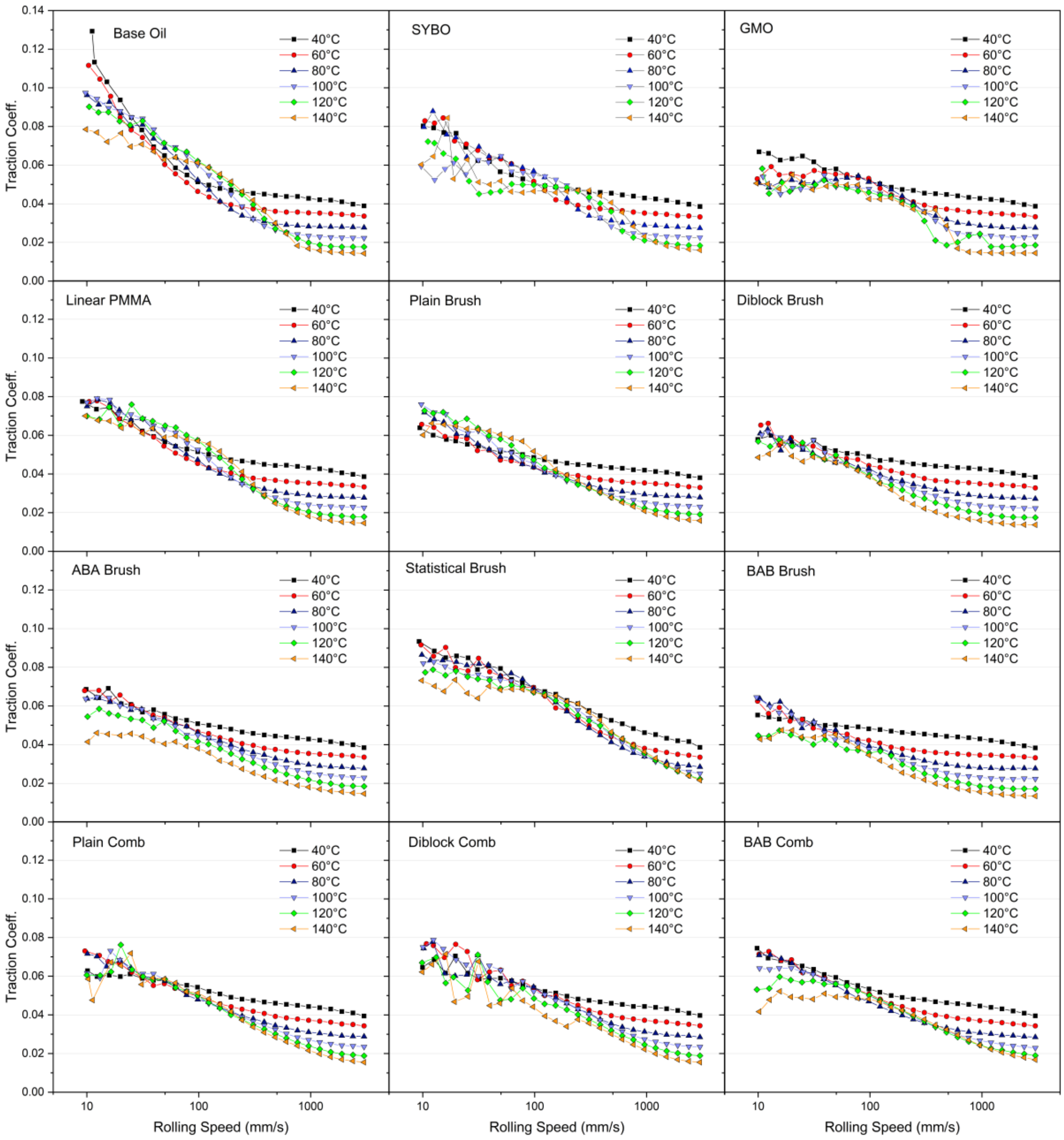

Figure S16. MTM testing of each compound performed at 1 wt. % additive in mineral oil, plotted for each temperature ranging from 40-140 °C.

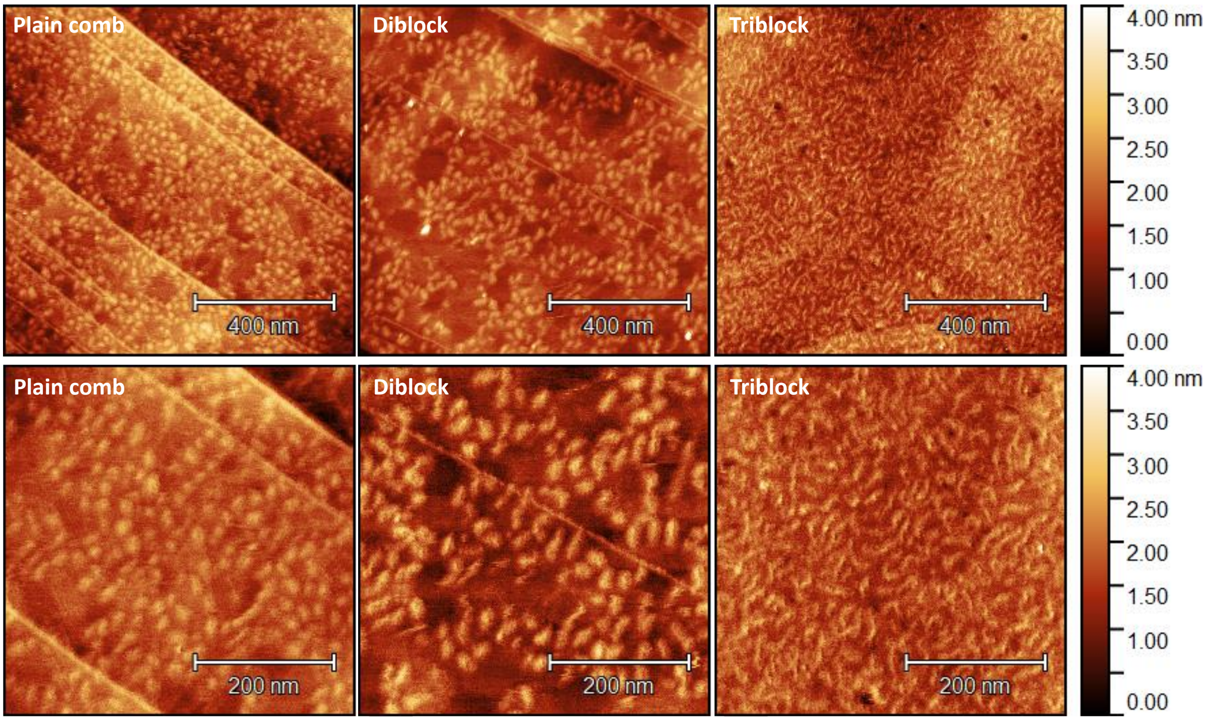

Figure S17: AFM imaging of comb compounds deposited onto HOPG substrate from dilute solution in chloroform.

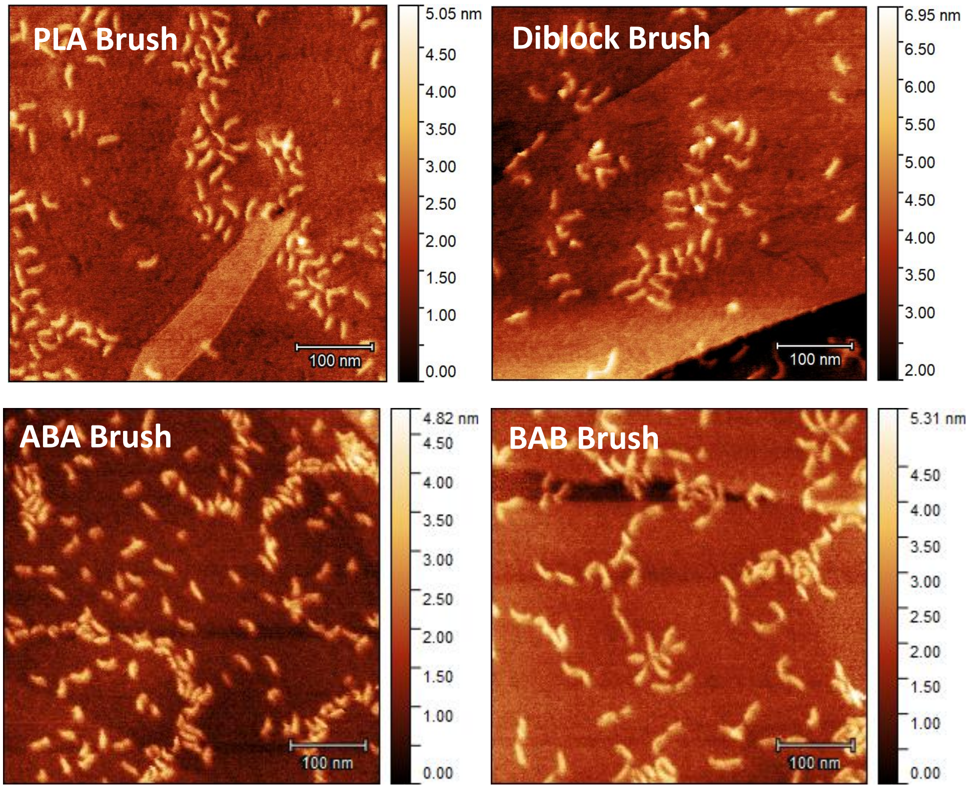

Figure S18: AFM images of bottlebrush compounds prepared from solutions (0.05 mg ml^{-1} CHCl3) drop casted onto a freshly cleaved HOPG substrate. Compared to the above comb compounds, the molecular size is better defined and a more cylindrical conformation is apparent.

4 References

1. Ferguson, C. J.; Hughes, R. J.; Nguyen, D.; Pham, B. T.; Gilbert, R. G.; Serelis, A. K.; Such, C. H.; Hawkett, B. S., Ab Initio Emulsion Polymerization by RAFT-Controlled Self-Assembly *Macromolecules* **2005,** *38* (6), 2191-2204.

2. Kerr, A.; Hartlieb, M.; Sanchis, J.; Smith, T.; Perrier, S., Complex Multiblock Bottle-Brush Architectures by RAFT Polymerization. *Chemical Communications* **2017,** *53* (87), 11901-11904.

3. Arnold, O.; Bilheux, J. C.; Borreguero, J. M.; Buts, A.; Campbell, S. I.; Chapon, L.; Doucet, M.; Draper, N.; Ferraz Leal, R.; Gigg, M. A.; Lynch, V. E.; Markvardsen, A.; Mikkelson, D. J.; Mikkelson, R. L.; Miller, R.; Palmen, K.; Parker, P.; Passos, G.; Perring, T. G.; Peterson, P. F.; Ren, S.; Reuter, M. A.; Savici, A. T.; Taylor, J. W.; Taylor, R. J.; Tolchenov, R.; Zhou, W.; Zikovsky, J., Mantid—Data analysis and visualization package for neutron scattering and μ SR experiments. *Nuclear Instruments and Methods in Physics Research Section A: Accelerators, Spectrometers, Detectors and Associated Equipment* **2014,** *764*, 156-166.

4. Breßler, I.; Kohlbrecher, J.; Thünemann, A. F., SASfit: a tool for small-angle scattering data analysis using a library of analytical expressions. *Journal of Applied Crystallography* **2015,** *48* (5), 1587–1598.

5. Kholodenko, A. L., Analytical calculation of the scattering function for polymers of arbitrary flexibility using the Dirac propagator. *Macromolecules* **1993,** *26* (16), 4179-4183.

6. Pedersen, J. S., Form factors of block copolymer micelles with spherical, ellipsoidal and cylindrical cores. *Journal of Applied Crystallography* **2000,** *33* (3), 637-640.

7. Clifton, L. A.; Hall, S. C. L.; Mahmoudi, N.; Knowles, T. J.; Heinrich, F.; Lakey, J. H., *Structural Investigations of Protein–Lipid Complexes Using Neutron Scattering*. Springer: New York, 2019.

8. Braun, L.; Uhlig, M.; von Klitzing, R.; Campbell, R. A., Polymers and surfactants at fluid interfaces studied with specular neutron reflectometry. *Advances in Colloid and Interface Science* **2017,** *247*, 130-148.

9. Wood, M. H.; Welbourn, R. J. L.; Charlton, T.; Zarbakhsh, A.; Casford, M. T.; Clarke, S. M., Hexadecylamine Adsorption at the Iron Oxide–Oil Interface. *Langmuir* **2013,** *29* (45), 13735-13742.

10. Holt, S. A.; Le Brun, A. P.; Majkrzak, C. F.; McGillivray, D. J.; Heinrich, F.; Lösche, M.; Lakey, J. H., An ion-channel-containing model membrane: structural determination by magnetic contrast neutron reflectometry. *Soft Matter* **2009,** *5* (13), 2576-2586.

11. Clifton, L. A.; Holt, S. A.; Hughes, A. V.; Daulton, E. L.; Arunmanee, W.; Heinrich, F.; Khalid, S.; Jefferies, D.; Charlton, T. R.; Webster, J. R. P.; Kinane, C. J.; Lakey, J. H., An Accurate In Vitro Model of the E. coli Envelope. *Angewandte Chemie International Edition* **2015,** *54* (41), 11952-11955.

12. Chen, M.; Moad, G.; Rizzardo, E., Thiocarbonylthio end group removal from RAFT-synthesized polymers by a radical-induced process. *J. Polym. Sci., Part A: Polym. Chem.* **2009,** *47* (23), 6704-6714.
